# Multiple sodium channel isoforms mediate the pathological effects of Pacific ciguatoxin-1

**DOI:** 10.1038/srep42810

**Published:** 2017-02-22

**Authors:** Marco C. Inserra, Mathilde R. Israel, Ashlee Caldwell, Joel Castro, Jennifer R. Deuis, Andrea M. Harrington, Angelo Keramidas, Sonia Garcia-Caraballo, Jessica Maddern, Andelain Erickson, Luke Grundy, Grigori Y. Rychkov, Katharina Zimmermann, Richard J. Lewis, Stuart M. Brierley, Irina Vetter

**Affiliations:** 1 Centre for Pain Research, Institute for Molecular Bioscience, The University of Queensland, 306 Carmody Rd, St Lucia, Queensland 4072, Australia; 2 Visceral Pain Group, South Australian Health and Medical Research Institute (SAHMRI), School of Medicine, Flinders University, Adelaide, South Australia 5000, Australia; 3 Centre for Nutrition and Gastrointestinal Diseases, Discipline of Medicine, University of Adelaide, South Australian Health and Medical Research Institute (SAHMRI), North Terrace, Adelaide, South Australia 5000, Australia; 4 Queensland Brain Institute, The University of Queensland, St Lucia, Queensland 4072, Australia; 5 Klinik für Anästhesiologie am Universitätsklinikum Erlangen, Friedrich-Alexander Universität Erlangen-Nürnberg, Erlangen, Germany; 6 School of Pharmacy, The University of Queensland, 20 Cornwall St, Woolloongabba, Queensland 4102, Australia

## Abstract

Human intoxication with the seafood poison ciguatoxin, a dinoflagellate polyether that activates voltage-gated sodium channels (Na_V_), causes ciguatera, a disease characterised by gastrointestinal and neurological disturbances. We assessed the activity of the most potent congener, Pacific ciguatoxin-1 (P-CTX-1), on Na_V_1.1–1.9 using imaging and electrophysiological approaches. Although P-CTX-1 is essentially a non-selective Na_V_ toxin and shifted the voltage-dependence of activation to more hyperpolarising potentials at all Na_V_ subtypes, an increase in the inactivation time constant was observed only at Na_V_1.8, while the slope factor of the conductance-voltage curves was significantly increased for Na_V_1.7 and peak current was significantly increased for Na_V_1.6. Accordingly, P-CTX-1-induced visceral and cutaneous pain behaviours were significantly decreased after pharmacological inhibition of Na_V_1.8 and the tetrodotoxin-sensitive isoforms Na_V_1.7 and Na_V_1.6, respectively. The contribution of these isoforms to excitability of peripheral C- and A-fibre sensory neurons, confirmed using murine skin and visceral single-fibre recordings, reflects the expression pattern of Na_V_ isoforms in peripheral sensory neurons and their contribution to membrane depolarisation, action potential initiation and propagation.

Ciguatera is the most common non-bacterial form of fish-borne illness and is caused by the consumption of fish contaminated with ciguatoxins^1^. These polycyclic ethers are lipophilic, heat stable ichthyotoxins that originate from benthic dinoflagellates of the genus *Gambierdiscus* that bloom in tropical and sub-tropical oceans around the world. Consumption of coral and seaweed contaminated with *Gambierdiscus* by herbivorous fish leads to bioaccumulation of the ciguatoxins through the food chain *via* larger carnivorous fish that in turn are consumed by humans and cause ciguatera. The communities most affected by ciguatera are those that rely on fish as a major part of their diet such as the island nations of the Pacific and Indian Oceans and the Caribbean Sea^2^,^3^,^4^. However, as the world’s oceans warm and algal blooms become more frequent, ciguatera is now emerging as a significant issue in Asia, America and parts of Europe^5^,^6^,^7^,^8^.

The presenting symptoms of ciguatera are predominantly neurotoxic in the majority of cases and include post-ingestion paraesthesiae, dysaesthesiae and heightened nociception, including the pathognomonic symptom of cold allodynia, which is associated with intense discomfort on exposure to cool temperatures^1^,^9^,^10^,^11^. In addition, early signs of ciguatera include gastrointestinal symptoms such as diarrhoea, vomiting and abdominal pain as well as musculoskeletal symptoms, in particular weakness and fatigue^1^. Cardiovascular symptoms such as bradycardia occur more rarely and often in more severe cases of poisoning^12^,^13^,^14^,^15^. This diverse symptomatology is believed to be caused by the interaction of the ciguatoxins with site 5 of the voltage-gated sodium channels (Na_V_)^11^,^16^,^17^,^18^. Mammalian Na_V_ channels Na_V_1.1–1.9 comprise a voltage-sensitive pore-forming α-subunit, which may also be associated with one of four auxiliary β-subunits that can modify the gating and kinetic profile of the α-subunit. The α-subunit consists of four homologous domains each containing six transmembrane segments that form the pore and facilitate voltage-sensing and ligand binding. Pacific ciguatoxin-1 (P-CTX-1), the most potent ciguatoxin congener thought to be responsible for the majority of symptoms associated with ciguatera in the Pacific^19^, elicits varied effects on the electrophysiological properties of Na_V_ channels and as a consequence enhances neuronal excitability. In dorsal root ganglion (DRG) neurons, P-CTX-1 shifts the voltage of activation of tetrodotoxin-sensitive (TTX-s) Na_V_ channels to more hyperpolarised potentials and decreases peak tetrodotoxin-resistant (TTX-r) Na^+^ current^20^. Similarly, in parasympathetic neurons P-CTX-1 enhances the open probability of single TTX-sensitive Na_V_ channels without altering the unitary conductance or reversal potential^21^. However, while it is known that the ciguatoxins modulate activity of TTX-s and TTX-r Na_V_ isoforms, the relative selectivity for and pharmacological effects on individual Na_V_ isoforms, and their association with diverse pathophysiological consequences have not been assessed to-date. Thus, the aims of the present study were to determine the relative activity and subtype-selectivity of the most potent congener of the ciguatoxins, P-CTX-1, on the mammalian Na_V_ isoforms Na_V_1.1–1.9 and to define the Na_V_ isoforms that contribute to the *in vivo* symptomatology associated with ciguatera.

## Results

### Subtype-selectivity of P-CTX-1 at Na_V_1.1–1.9

**Figure 1:**
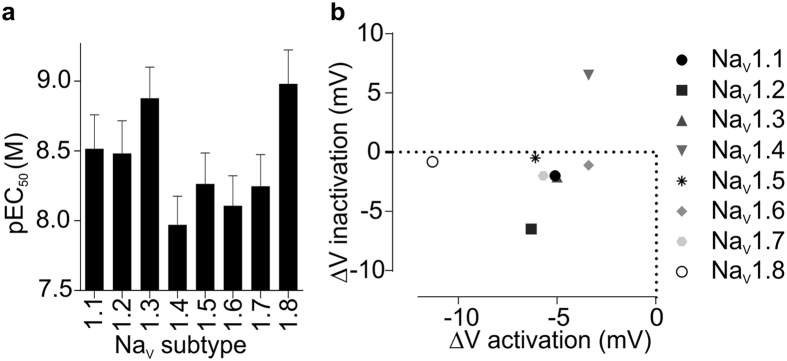
P-CTX-1 subtype selectivity assessed using a high-throughput membrane potential FLIPR^Tetra^ assay. (**a**) P-CTX-1 concentration-dependently potentiated veratridine-induced responses in mammalian cells heterologously expressing Na_V_1.1–Na_V_1.8 with little subtype selectivity. (**b**) P-CTX-1 (10 nM) induced shifts in the voltage of activation at Na_V_1.1–Na_V_1.8 and the voltage-dependence of inactivation at Na_V_1.2, Na_V_1.3 and Na_V_1.4. Data are presented as mean ± SEM from n = 3–6 independent experiments.

Although ciguatoxin is known to affect TTX-sensitive and TTX-resistant Na_V_ channels^20^, the specific effects of P-CTX-1 on individual Na_V_ subtypes have not previously been reported. We first assessed the relative potency and selectivity of P-CTX-1 for Na_V_ isoforms using a high-throughput membrane potential assay^22^. In HEK293 cells stably expressing hNa_V_1.1–1.8, P-CTX-1 elicited a concentration-dependent potentiation of Na_V_-mediated responses with little subtype-selectivity (Fig. 1a; EC_50_ ± SEM: Na_V_1.1, 7.9 ± 3.1 nM; Na_V_1.2, 8.3 ± 3.9 nM; Na_V_1.3, 3.4 ± 1.6 nM; Na_V_1.4, 18.4 ± 6.9 nM; Na_V_1.5, 10.8 ± 3.8 nM; Na_V_1.6, 18.1 ± 9.4 nM; Na_V_1.7, 13.0 ± 5.8 nM; Na_V_1.8, 2.1 ± 0.7 nM). Overall, Na_V_1.3 and Na_V_1.8 were most potently affected, while Na_V_1.4 was the isoform least potently affected, although the relative selectivity for these isoforms was not statistically significant (ANOVA, *p* > 0.05). These results indicate that P-CTX-1 is a potent but relatively non-specific modulator of Na_V_ channels. Therefore, we sought to assess the effects of P-CTX-1 on the electrophysiological properties of hNa_V_1.1–1.9 in more detail.

### Differential effects of P-CTX-1 on the electrophysiological parameters of Na_V_1.1–1.9

**Figure 2:**
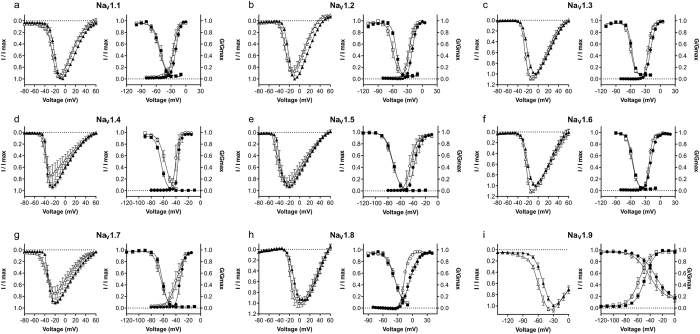
Effects of P-CTX-1 on the electrophysiological parameters of Na_V_1.1–1.9 assessed by automated whole-cell patch clamping in mammalian cells. P-CTX-1 (white triangles) caused a leftward shift in the I-V curves at all Na_V_ subtypes compared to control (black triangles). P-CTX-1 (white circles) significantly shifted the V_1/2_ of voltage-dependence of activation to more hyperpolarised potentials at all Na_V_ subtypes compared to control (black circles). P-CTX-1 (white squares) only caused a significant shift in the V_1/2_ of voltage-dependence of steady-state fast inactivation at Na_V_1.2, Na_V_1.3, Na_V_1.4 and Na_V_1.9 compared to control (black squares). I_max_ is the maximal peak amplitude, determined for each cell, in the absence of the toxin. Data are presented as mean ± SEM, with n = 4–10 cells per data point.

Using whole cell patch-clamp recordings, we further assessed the effects of P-CTX-1 on the electrophysiological parameters of hNav1.1–1.9 expressed heterologously in HEK293 cells (Figs 1b and 2 and Table 1). The effect of P-CTX-1 on peak current was voltage-dependent, causing an increase in peak current at more hyperpolarised potentials at all subtypes (Fig. 2), with maximal peak current significantly increased only for Na_V_1.6 (Table 1). Accompanying the voltage-dependent changes in peak current, P-CTX-1 significantly shifted the V_1/2_ for the voltage-dependence of activation to more hyperpolarising potentials at all Na_V_ subtypes (ΔV_1/2_ activation: Na_V_1.1, −5.1 mV; Na_V_1.2, −6.3 mV; Na_V_1.3, −5.0 mV; Na_V_1.4, −3.4 mV; Na_V_1.5, −6.1 mV; Na_V_1.6, −3.4 mV; Na_V_1.7, −5.7 mV; Na_V_1.8, −11.3 mV; Na_V_1.9, −8.9 mV; Figs 1b and 2 and Table 1). These effects are consistent with Na_V_ channel activator activity and were not observed in time-matched controls. Interestingly, P-CTX-1 also significantly changed the slope factor of the conductance-voltage curves for Na_V_1.3, Na_V_1.6, Na_V_1.7 and Na_V_1.8 (Fig. 2 and Table 1). For Na_V_1.3, Na_V_1.6 and Na_V_1.8, the slope factor decreased, indicating higher conductance at more depolarised membrane potentials, whilst for Na_V_1.7 the slope factor increased, indicating higher conductance at more hyperpolarised membrane potentials.

**Figure 3:**
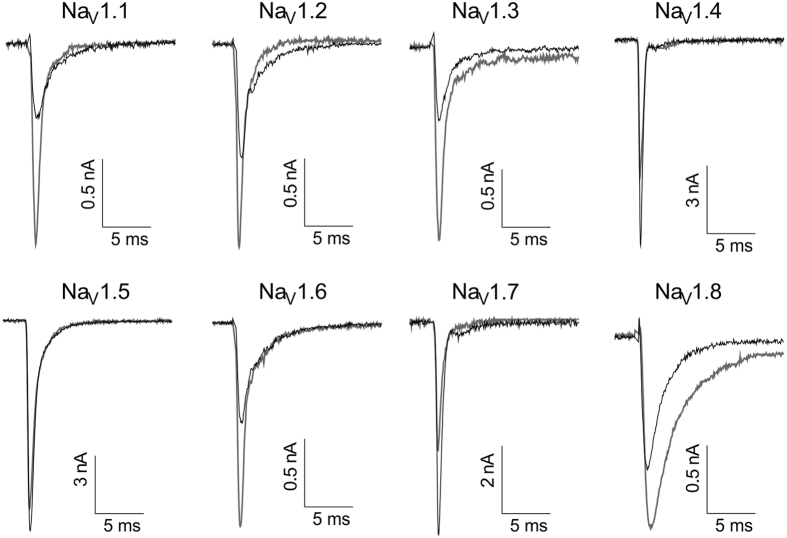
Representative effects of P-CTX-1 on Na_V_1.1–1.8 currents assessed by automated whole-cell patch clamping in mammalian cells. Currents were elicited using a holding potential of −80 mV, followed by a pre-pulse of −100 mV for 50 ms and then a 50 ms pulse of −20 mV for Na_V_1.1–1.7 and +10 mV for Na_V_1.8. Black traces represent control current and grey traces represent current in the presence of P-CTX-1.

The effects of P-CTX-1 on the voltage-dependence of steady-state fast inactivation were less profound. A significant hyperpolarising shift was observed in the V_1/2_ for Na_V_1.2, Na_V_1.3 and Na_V_1.9, with a significant depolarising shift occurring only at Na_V_1.4 (ΔV_1/2_ inactivation: Na_V_1.1, −2.0 mV; Na_V_1.2, −6.9 mV; Na_V_1.3, −2.1 mV; Na_V_1.4, + 6.5 mV; Na_V_1.5, −0.5 mV; Na_V_1.6, −1.1 mV; Na_V_1.7, −2.0 mV; Na_V_1.8, −0.8 mV; Na_V_1.9, −10.6 mV; Fig. 2 and Table 1). P-CTX-1 had differential effects on Na_V_ channel activation and inactivation kinetics, including significantly decreasing the time to peak at Na_V_1.2, significantly decreasing the inactivation time constant for Na_V_1.1, Na_V_1.2 and Na_V_1.3, and increasing the inactivation time constant for Na_V_1.8 (Fig. 3 and Table 2). P-CTX-1 also produced a significant persistent current at Na_V_1.3 (I/I_max_, 3.4 ± 0.8%) and Na_V_1.8 (I/I_max_, 7.6 ± 0.2%).

### P-CTX-1 reduces the activation threshold of single Na_V_1.8 and Na_V_1.7 channels and lengthens their active periods

Our whole-cell measurements indicated that P-CTX-1 lowers the threshold for activation of Na_V_1.7 and Na_V_1.8, two key isoforms contributing to action potential generation and propagation in nociceptive neurons (Fig. 2, Table 1). However, in the presence of P-CTX-1, currents mediated by Na_V_1.8 channels inactivated with a slower time constant than those mediated by Na_V_1.7 (Fig. 3, Table 2). We thus used single channel recordings to further investigate how P-CTX-1 potentiated Na_V_1.7 and Na_V_1.8 by measuring single channel current amplitude and the duration of single channel active periods (activations).

**Figure 4:**
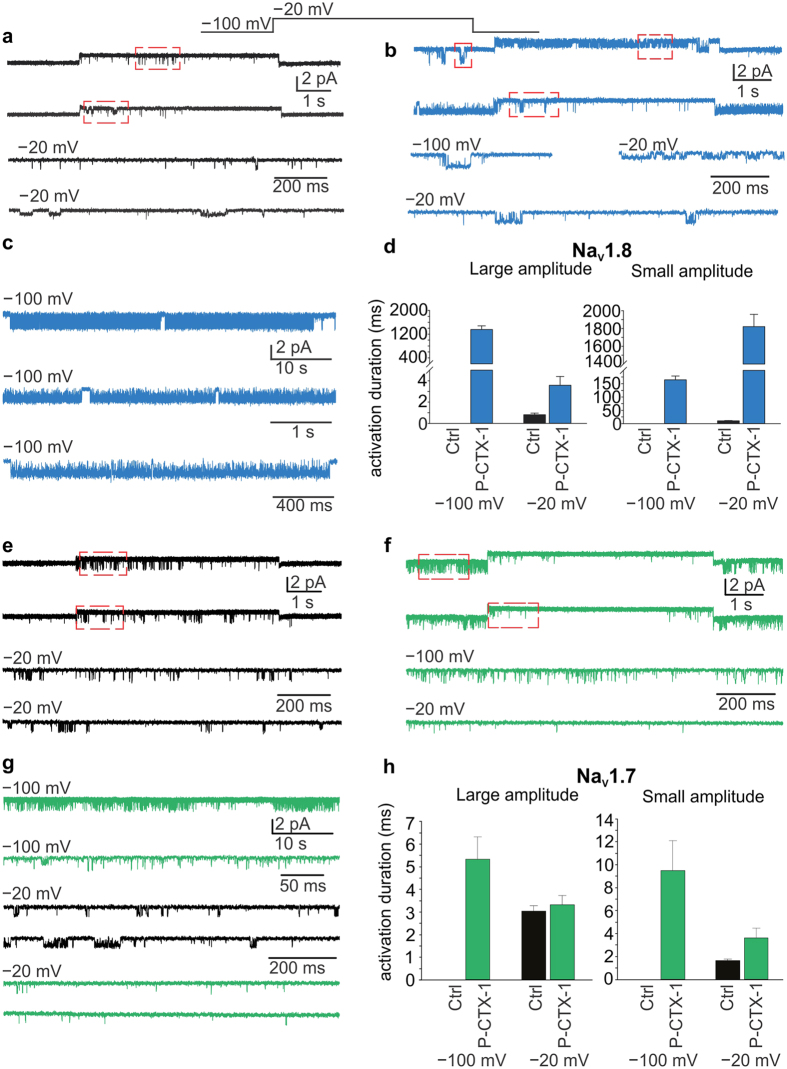
Effect of P-CTX-1 on single channel activity of Na_V_1.7 and Na_V_1.8. (**a**) Single channel activity recorded from a membrane patch expressing Nav 1.8 channels after depolarising the membrane from −100 mV to −20 mV (shown above). Two main amplitudes were apparent, but only at −20 mV. These are boxed in red and shown on an expanded time scale below. (**b**) A similar activation protocol was applied to a recorded patch after exposing it to 1 nM P-CTX-1 for 10 minutes. Note the presence of activity at −100 mV and −20 mV of both amplitude levels. Areas boxed in red are shown below on an expanded time scale. (**c**) Continuous recording from a patch exposed to 1 nM P-CTX-1 for 10–12 minutes at −100 mV showing long activation periods, interrupted by brief inactivity, likely corresponding to channel inactivation. The two lower current traces are from the same patch but on an expanded time scale. (**d**) Summary plots of the mean durations of activations before (Ctrl) and after (P-CTX-1) exposure to P-CTX-1 for activations at both amplitude levels. (**e**) Single channel recordings from a patch expressing Na_V_ 1.7 channels, showing negligible activity at −100 mV, but significant activity at −20 mV. The sections boxed in red are shown below at a higher temporal resolution. (**f**) After exposing the recorded path to 1 nM P-CTX-1 for 10 minutes the channels exhibited frequent openings at −100 mV, but less activity at −20 mV. Selected sections are boxed in red and shown at higher temporal resolution below. (**g**) Sample currents after exposure to P-CTX-1 at −100 mV at two temporal resolutions (above) and control sweeps (black) and after P-CTX-1 exposure (green) at −20 mV, showing a reduction in channel activity. (**h**) Summary plots of the mean durations of activations before (Ctrl) and after (P-CTX-1) exposure to P-CTX-1 for activations at both amplitude levels.

Excised outside-out membrane patches expressing Na_V_1.8 channels were subjected to an activating voltage step of −100 mV to −20 mV (Fig. 4). At −100 mV, channel activations were too infrequent for any reliable analysis (Fig. 4a). However, depolarising the patch to −20 mV elicited brief bursts of single Na_V_1.8 channel openings. Multiple amplitude levels were discernible at −20 mV, the predominant ones being a large (1.36 ± 0.03 pA; duration 0.8 ± 0.2 ms) and a small (0.63 ± 0.04 pA; duration 10.5 ± 1.0 ms) amplitude opening. After exposure to P-CTX-1 (1 nM), significant channel activity at both amplitude levels was observed at −100 mV and −20 mV (Fig. 4b). The two amplitudes were slightly larger at −100 mV (1.64 ± 0.08 and 0.69 ± 0.05 pA) than at −20 mV (1.43 ± 0.04 pA and 0.63 ± 0.02 pA), reflecting the difference in driving force (in the absence of Na^+^ in the pipette solution), but were essentially unchanged in the presence of P-CTX-1. However, P-CTX-1 did produce dramatic effects on the duration of single channel activations (Fig. 4b and c). At −100 mV, the mean duration of the large channel activations was substantially longer (1357 ± 122 ms) than at −20 mV (3.6 ± 0.8 ms), lasting over 5 s in some patches (Fig. 4c). Conversely, the duration of the smaller amplitude activations were enhanced more at −20 mV (1821 ± 138 ms) than at −100 mV (165 ± 14 ms; Fig. 4d).

The above experiments were repeated on excised membrane patches expressing Na_V_1.7 channels. At −100 mV, Na_V_1.7 channels were mostly inactive, whereas stepping the membrane potential to −20 mV elicited short-lived channel openings (Fig. 4e) of similar amplitude to those mediated by Na_V_1.8 channels. Again, two main amplitudes were discernible. The larger amplitude was predominant (>90% of openings, amplitude 1.28 ± 0.03 pA, duration 3.1 ± 0.2 ms), whereas the smaller amplitude openings were rarely observed (<10% of openings, amplitude 0.74 ± 0.03 pA, duration 1.6 ± 0.1 ms). After exposure of the recorded patches to P-CTX-1, significant activity was observed at −100 mV (Fig. 4f and g), however, at −20 mV the frequency of openings was markedly reduced compared with control (Fig. 4f). As for the Na_V_1.8 channels, both the larger and smaller Na_V_1.7 amplitude openings were relatively unaffected by P-CTX-1 at either −100 mV (larger amplitude, 1.67 ± 0.03 pA; smaller amplitude, 0.87 ± 0.03 pA) or −20 mV (larger amplitude, 1.31 ± 0.04 pA; smaller amplitude 0.75 ± 0.03 pA). In contrast to Na_V_1.8, P-CTX-1 had relatively minor effects on the mean durations of Na_V_1.7 single channel activity, which were increased for larger (5.3 ± 0.9 ms mean duration) and smaller (9.5 ± 2.5 ms mean duration) amplitudes at −100 mV. At −20 mV the mean durations for large and small amplitude openings were 3.3 ± 0.4 ms and 3.6 ± 0.9 ms, respectively (Fig. 4h).

The single channel experiments confirmed that the effects of P-CTX-1 on Na_V_1.8 substantially differ from effects on Na_V_1.7. The most apparent common effect of P-CTX-1 on Na_V_1.7 and Na_V_1.8 was a decrease of the threshold of activation to a level where the channels are not normally active (−100 mV). This effect also manifests in the whole-cell data, with a decrease in the V_1/2_ for activation for both subtypes (Table 1). Although P-CTX-1 generally lengthened the periods of discrete single channels activations in both subtypes, the effect on Na_V_1.8 was substantially greater. The P-CTX-1-induced increase in the duration of the activations likely underlies the significant increase in the inactivation time constant of ensemble currents (Table 2).

### Contribution of Na_V_ isoforms to P-CTX-1-induced responses of sensory dorsal root ganglion neurons

**Figure 5:**
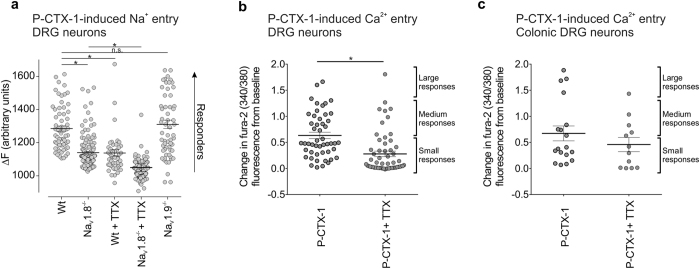
P-CTX-1 induced responses in isolated dorsal root ganglion neurons. (**a**) Increases in intracellular Na^+^ in response to P-CTX-1 (1 nM) were assessed in dissociated DRG neurons from wild-type, Na_V_1.8^−/−^ and Na_V_1.9^−/−^ mice in the absence and presence of TTX (1 μM). P-CTX-1-induced responses were significantly (*P < 0.0001) decreased in DRGs from Na_V_1.8^−/−^, but not Na_V_1.9 ^−/−^ mice and after treatment with TTX. Data is presented as mean ± SEM from n = 51–163 cells/group. (**b**) Increases in intracellular Ca^2+^ in response to P-CTX-1 (1 nM) were assessed in dissociated DRG neurons from wild-type mice in the absence and presence of TTX (1 μM). P-CTX-1-induced responses were significantly decreased in DRG neurons co-incubated with TTX (*P < 0.0001). Data is presented as mean ± SEM from n = 49 neurons (P-CTX-1) and n = 57 neurons (P-CTX-1 + TTX). (**c**) P-CTX-induced activation of intracellular Ca^2+^ within identified colonic DRG neurons. Note that 1 nM P-CTX-1 induced a different magnitude of responses (small, medium and large). TTX (0.5 μM) reduced P-CTX-1-induced activation of colonic DRG neurons. This reduction was specially marked in colonic neurons exhibiting large responses when co-incubated with TTX. Data is presented as mean ± SEM from n = 18 colonic DRG neurons (P-CTX-1) and n = 12 colonic DRG neurons (P-CTX-1 + TTX). *P < 0.0001 using One-way ANOVA (**a**) or student’s t-test (**b**,**c**).

As individual sensory neurons can express multiple Na_V_ isoforms, we next assessed the relative contribution of TTX-s and TTX-r Na_V_ isoforms to P-CTX-1-induced responses using fluorescent live cell imaging of cultured dorsal root ganglion (DRG) neurons. At a concentration of 5 nM, P-CTX-1 elicited Na^+^ influx in most DRG neurons, albeit the size of the responses varied considerably (Fig. 5a). Consistent with the observed effect on the electrophysiological properties of Na_V_1.8, P-CTX-induced Na^+^ influx was significantly decreased in DRG neurons from Na_V_1.8^−/−^ animals (Control (ΔF), 1285 ± 15; Na_V_1.8^−/−^ (ΔF), 1140 ± 8; *p* < 0.001, Fig. 5a). Nevertheless, many individual cells still responded with large increases in intracellular Na^+^ to application of P-CTX-1. Similarly, treatment of DRG neurons with TTX (1 μM) significantly decreased P-CTX-1-induced responses compared with control, although several cells with large responses remained, supporting an important contribution of both TTX-s and TTX-r isoforms to the enhanced sensory neuronal excitability induced by P-CTX-1 (Control (ΔF), 1285 ± 15; TTX (ΔF), 1138 ± 19; *p* < 0.001, Fig. 5a). Exposure of DRG neurons from Na_V_1.8^−/−^ animals to TTX (1 μM) further decreased responses (Na_V_1.8^−/−^ + TTX (ΔF), 1050 ± 23, *p* < 0.01, Fig. 5a), although some small responses remained. Whilst overall, Na^+^ influx was not significantly changed in DRGs from Na_V_1.9^−/−^ animals (Na_V_1.9^−/−^ (ΔF), 1310 ± 25, *p* > 0.01, Fig. 5a), the presence of small P-CTX-1-evoked responses in Na_V_1.8^−/−^ DRG neurons treated with TTX indicates that Na_V_1.9 contributes to P-CTX-1-induced Na^+^ influx in some cells (Fig. 5a).

As a secondary process to Na^+^ channel activation, we also assessed P-CTX-1 induced Ca^2+^ influx in DRG neurons. Similar to what we observed with Na^+^ entry, P-CTX-1 (1 nM) elicited Ca^2+^ influx in most DRG neurons with considerable variability in the size and magnitude of the responses (Fig. 5b). Treatment with TTX (1 μM) significantly decreased P-CTX-1-induced Ca^2+^ responses compared with control P-CTX-1 response (*p < 0.0001), although several large responses remained. This further supports an important contribution of both TTX-s and TTX-r isoforms to the enhanced sensory neuronal excitability induced by P-CTX-1.

As the early signs of ciguatera include gastrointestinal symptoms such as abdominal pain, we tested P-CTX-1 on DRG neurons that specifically innervate the colon. We found that P-CTX-1-induced Ca^2+^ responses were qualitatively similar in colonic sensory neurons to those in non-colonic neurons (Fig. 5c), with 83.3% (15/18) of colonic DRG neurons activated by P-CTX-1. The size of the responses also varied considerably including small (46.8% of all activated neurons), medium (26.7% of all activated neurons) and large (26.7% of all activated neurons) magnitude responses (Fig. 5c). TTX (0.5 μM) reduced the number of colonic DRG neurons activated by P-CTX-1 to 58.3% (7/12), and also reduced the magnitude of Ca^2+^ influx induced by P-CTX-1 (Fig. 5c). The P-CTX-1-induced large magnitude responses in colonic neurons appeared to be predominantly mediated by TTX-s channels (Fig. 5c), as only 5% (1 out of 18) of colonic DRG neurons responded to P-CTX-1 when co-incubated with TTX compared with 33% (4 out 12) of colonic DRG neurons with large responses induced by P-CTX-1 alone.

### P-CTX-1 evokes action potential firing in dermal and colonic sensory afferents

To further pharmacologically explore the contribution of individual Na_V_ subtypes to enhanced excitability induced by P-CTX-1, we recorded propagated action potentials from single receptive fields of sensory afferents, recorded extracellularly from murine skin-saphenous nerves and from colonic splanchnic nerves.

**Figure 6:**
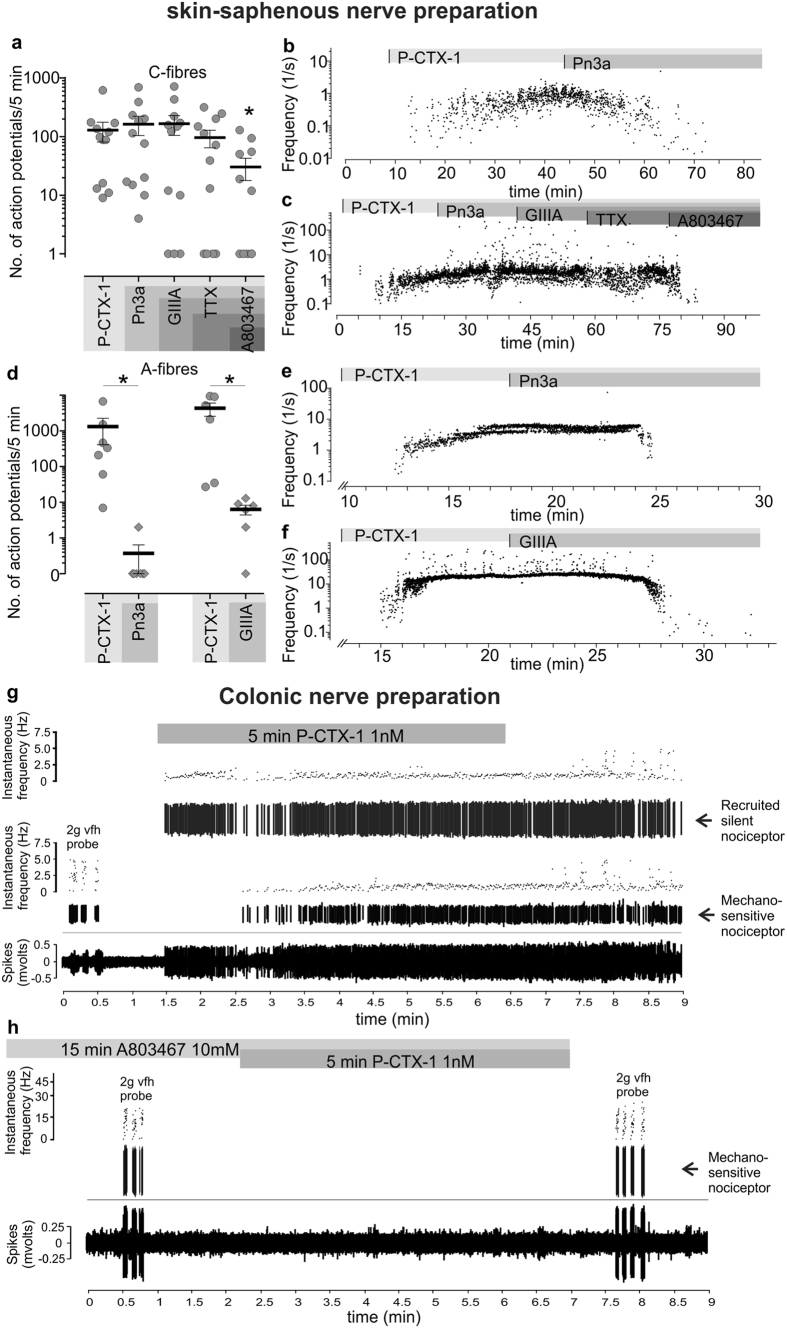
P-CTX-1 induces spontaneous action potential firing in peripheral sensory afferents. (**a**) Effect of P-CTX-1 (0.5 nM) on single C-fibres recorded from a skin-saphenous nerve preparation in the mouse. Selective inhibitors of Na_V_1.7 (Pn3a, 1 μM); Na_V_1.6 (GIIIA, 10 μM), TTX-s Na_V_ isoforms (TTX, 1 μM) and Na_V_1.8 (A803467, 10 μM) were sequentially perfused at the receptive field to assess cumulative effects of Na_V_ inhibition on action potential firing. Each data point represents the number of action potentials/5 min from a single C-fibre receptive field. (**b**) Representative recording of a C-fibre silenced by the selective Na_V_1.7 inhibitor Pn3a (1 μM). (**c**) Representative recording of a C-fibre unaffected by Pn3a (1 μM), GIIIA (10 μM) and TTX (1 μM) and silenced by the Na_V_1.8 inhibitor A803467 (10 μM). (**d**) Effect of P-CTX-1 (0.5 nM) on single A-fibres recorded from a skin-saphenous nerve preparation in the mouse. Selective inhibitors of either Na_V_1.7 (Pn3a, 1 μM) or Na_V_1.6 (GIIIA, 10 μM) almost completely silenced P-CTX-1-induced action potential firing in A-fibres. Each data point represents the number of action potentials/5 min from a single A-fibre receptive field. (**e**) Representative recording of an A-fibre silenced by Pn3a (1 μM) and (**f**) GIIIA (10 μM). (**b,c** and **e,f**) Each data point represents a single action potential and is plotted as a function of instantaneous frequency (1/s) to represent the time elapsed since the previous action potential. Arrows indicate time points of superfusion with compounds. (**g**) P-CTX-1 (1 nM) is a potent activator of colonic nociceptors (activating 14/19 afferents) and also recruits silent afferents (9/19 afferents). (**h**) Pre-incubation with the Na_V_1.8 blocker A803467 (10 μM for 15 min) prevents P-CTX-1-induced firing of colonic nociceptors (7/7 afferents tested). Data are presented as mean ± SEM. Statistical significance was determined using (a) One-way ANOVA with Friedman post-hoc test or (d) student’s matched observations t-test and defined as p < 0.05 (*).

In the murine skin-saphenous nerve preparation, P-CTX-1 (0.5 nM) induced action potential firing in all previously quiescent C fibres (12/12), with some fibres exhibiting a modest increase in activity (4/12 fibres; <50 action potentials/5 min) (Fig. 6a). Consistent with our previous findings^9^, action potential firing induced by P-CTX-1 in the skin was mediated predominantly by TTX-r Na_V_ isoforms in C-fibres, as treatment with the selective Na_V_1.7 inhibitor Pn3a^23^ (1 μM, Suppl Fig. 1), the Na_V_1.6 inhibitor GIIIA (10 μM), or TTX (1 μM) did not significantly decrease action potential firing overall (*p* > 0.05, Fig. 6a). However, some individual fibres (4/12) were silenced after inhibition of TTX-s Na_V_ isoforms (Fig. 6b). Notably, the P-CTX-1-induced firing frequency of these fibres was modest (action potentials/5 min: 53.2 ± 40.6) in comparison with fibres that were unaffected by TTX (action potentials/5 min: 166.1 ± 66.6). Similar to our previous findings using recordings from Na_V_1.8^−/−^ animals^9^, where P-CTX-1-induced firing in C-fibres was markedly decreased, the Na_V_1.8 inhibitor A803467 (10 μM; *p* < 0.05) significantly decreased action potential firing frequency by 62.8 ± 15.5% (Fig. 6c).

In contrast, P-CTX-1 elicited action potential firing in only 44.8% (13/29) of A-fibres, with responding fibres displaying significantly (*p* < 0.05) slower conduction velocities (6.28 ± 0.95 m/s, n = 6) than non-responders (10.64 ± 1.50 m/s, n = 5). Inhibition of the TTX-s isoforms Na_V_1.6 or Na_V_1.7 had a profound effect on action potential firing in A-fibres, with both Pn3a and GIIIA almost completely silencing P-CTX-1-induced activity (Fig. 6d). Interestingly, inhibition of either Na_V_1.7 (Fig. 6e) or Na_V_1.6 (Fig. 6f) appeared to be sufficient to virtually abolish spontaneous action potential firing, suggesting that both channels act in concert to maintain P-CTX-1-induced ongoing activity. Thus, both TTX-s and TTX-r isoforms contribute to the enhanced excitability of peripheral sensory neurons caused by P-CTX-1, albeit with their relative contributions differing between fibre types.

Unlike skin afferents, all colonic afferents have conduction velocities in the C-fibre range^24^,^25^. Using an *ex-vivo* colonic afferent preparation, we investigated the effects of P-CTX-1 on colonic nociceptive afferents that respond at noxious levels of distension or high intensity circular stretch and are therefore likely to signal “nociceptive” events^26^. We found that P-CTX-1 (1 nM) potently activated 74% of colonic nociceptors (14/19) as well as recruiting a population of ‘silent nociceptors’ (47%, 9/19 preparations) (Fig. 6g). Consistent with the results on C-fibres in the skin-nerve preparation, incubation with the Na_V_1.8 inhibitor A803467 (10 μM) abolished P-CTX-1-induced action potential firing in all colonic nociceptors tested (7/7) (Fig. 6h).

### Multiple sodium channel isoforms mediate the *in vivo* actions of P-CTX-1

We recently established an animal model of ciguatera based on the intraplantar administration of P-CTX-1 in mice and showed that ciguatoxin-induced cold allodynia is mediated through TRPA1-expressing C-fibres and involves Na_V_1.8^9^. However, whilst we have shown that both TTX-s and TTX-r Na_V_ isoforms contribute to increased neuronal excitability evoked by P-CTX-1, the relative contribution of Na_V_1.1–1.9 to the symptomatology of ciguatera remains unresolved. We thus sought to assess the *in vivo* contribution of individual Na_V_ isoforms to P-CTX-1-induced pain and gastrointestinal effects in relevant murine models.

### P-CTX-1-induced spontaneous dermal pain behaviours are mediated by Na_V_1.6 and Na_V_1.7

**Figure 7:**
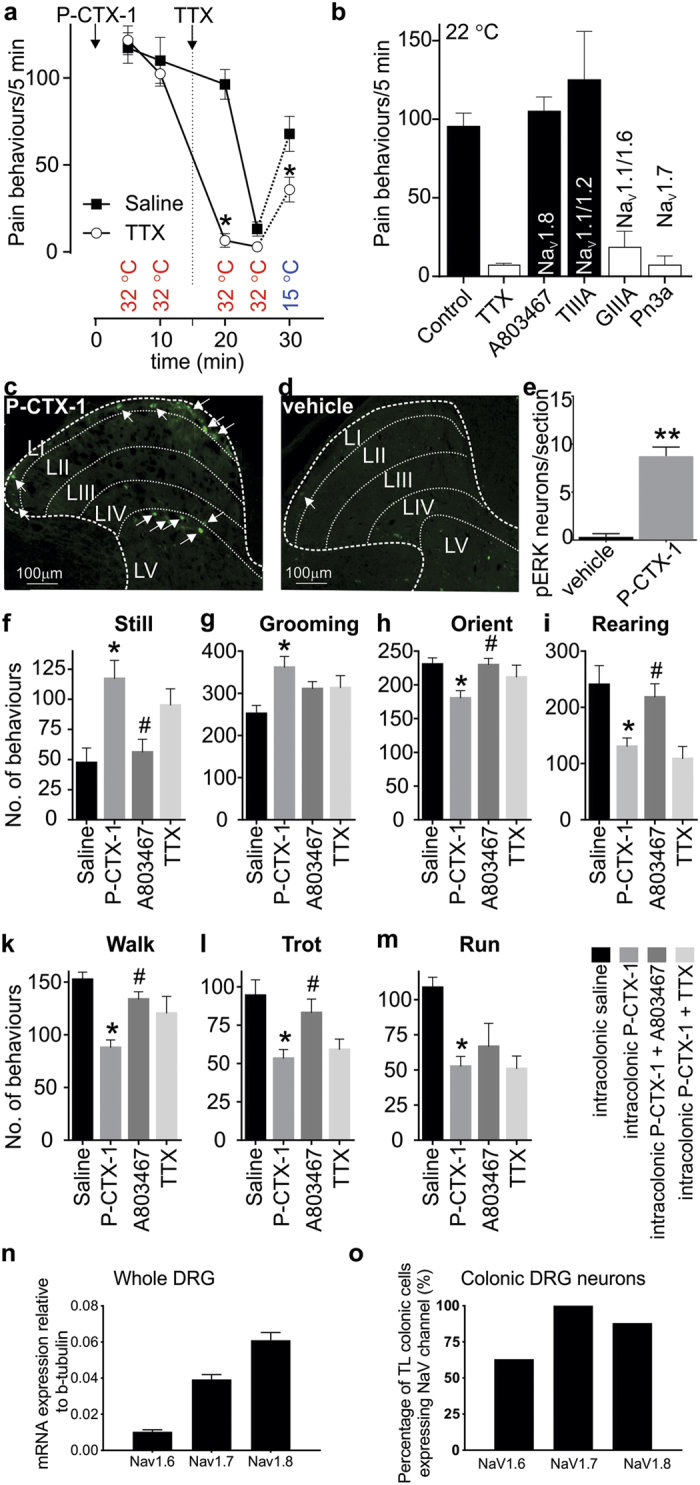
Multiple Na_V_ isoforms mediate the *in vivo* effects of P-CTX-1. (**a**) Intraplantar injection of P-CTX-1 in C57BL/6 mice causes spontaneous pain behaviours at 32 °C which are completely blocked by local administration of TTX (2 μM). Cold allodynia evoked at 15 °C is partially reduced by TTX. (n = 5/group) (**b**) At ambient temperature, P-CTX-1-induced nocifensive behaviours were significantly decreased by co-administration of TTX (1 μM), the selective Na_V_1.7 inhibitor Pn3a (3 μM) and the Na_V_1.6 inhibitor GIIIA (10 μM), but not the Na_V_1.8 inhibitor A803467 (10 μM) or the Na_V_1.1/1.2 inhibitor TIIIA (10 μM). (n = 4–12 animals/group). (**c**) *In vivo* intra-colonic administration of P-CTX-1 (1 nM) activates nociceptive endings within the wall of the colon, leading to signalling into the dorsal horn and activation of neurons within the thoracolumbar spinal cord, as identified by pERK immunoreactivity (arrows). pERK-IR neurons were predominantly located in the nociresponsive superficial dorsal horn (laminae I) and also in laminae V. (**d**) Intracolonic administration of vehicle alone did not induce dorsal horn neuron pERK immunoreactivity. (**e**) Group data showing that P-CTX-1 evoked significant dorsal horn neuron activation (**p < 0.005) compared with vehicle administered mice (n = 3–4 animals/group). (**f–m**) A novel mouse model of P-CTX-1-induced visceral pain. Intracolonic administration of P-CTX-1 (50 μl, 10 nM) caused a significant (*p < 0.05 compared with Saline control) increase in (**f**) still and (**g**) grooming behaviours as well as a significant (*p < 0.05 compared with Saline control) decrease in (**h**) orienting, (**i**) rearing, (**k**) walking, (**l**) trotting and (**m**) running. These behavioural changes were reversed by A803467 (100 mg/kg; ^#^p < 0.05 compared with P-CTX-1) but not intracolonic TTX (100 nM). (n = 6–12 animals/group) (**n**) Na_V_1.8 is the most highly expressed Na_V_ isoform in whole thoracolumbar DRG (n = 5 animals/target). (**o**) Na_V_1.8 is highly expressed within thoracolumbar DRG neurons innervating the colon (88%: 21 out of 24 colonic neurons). Data are presented as mean ± SEM. Statistical significance (defined as p < 0.05) was determined using student’s t-test (**a**,**e**) or One-way ANOVA with Sidak’s multiple comparison (**b**,**f–m**).

Cold allodynia is a pathognomonic symptom of ciguatera and occurs in up to 94% of ciguatera sufferers. However, ciguatera is also associated with a number of non-thermally evoked painful dysaesthesias attributable to the direct effects of ciguatoxin on Na_V_ channels expressed on peripheral sensory neurons, including arthralgias, myalgias, proctalgia, odontalgia, dysuria and dyspareunia. Indeed, intracutaneous administration of P-CTX-1 in humans elicits an axon reflex flare and spontaneous pain; effects that also occur after intraplantar administration of P-CTX-1 in mice^9^,^27^. In order to determine the Na_V_ isoforms contributing to the ciguatoxin-induced *in vivo* effects, we first assessed the effect of local administration of TTX (2 μM) on P-CTX-1-induced dermal pain behaviours. To enable assessment of TTX effects on both spontaneous pain behaviours as well as cold allodynia, we first administered P-CTX-1 (10 nM; 20 μl) by intraplantar injection and assessed nocifensive behaviours at 32 °C for 10 min. A subsequent intraplantar injection of TTX (2 μM, 20 μl; 6.6 ± 3.9 flinches/5 min) almost completely reversed spontaneous pain behaviours compared with intraplantar injection of saline (20 μl; 96.2 ± 8.5 behaviours/5 min) but only partially reversed cold allodynia assessed at 15 °C (Saline, 67.8 ± 10.1 behaviours/5 min; TTX, 35.8 ± 7.1 behaviours/5 min) (Fig. 7a). Consistent with the profound effect of TTX, which was also observed when co-administered with P-CTX-1 (5 nM, 40 μl, 95.6 ± 9.1 behaviours/5 min; TTX (1 μM), 7.1 ± 1.3 behaviours/5 min; *p* < 0.01), treatment with the Na_V_1.8 inhibitor A803467 (10 μM, 105.7 ± 9.4 behaviours/5 min) had no effect on P-CTX-1-induced pain behaviours (Fig. 7b). Similarly, P-CTX-1-induced pain behaviours were also unchanged after co-administration of the Na_V_1.1/1.2 inhibitor TIIIA (10 μM; 125.7 ± 31.1 behaviours/5 min), while intraplantar administration of the Na_V_1.6 inhibitor GIIIA (10 μM; 19.0 ± 10.1 behaviours/5 min; *p* < 0.01) significantly reduced P-CTX-1-induced pain behaviours (Fig. 7b). In addition, the selective Na_V_1.7 inhibitor Pn3a (3 μM; 7.7 ± 5.7 behaviours/5 min; *p* < 0.01) was also effective at reducing ciguatoxin-induced pain behaviours (Fig. 7b). Overall, these data suggest that the P-CTX-1 effects on TTX-s Na_V_ isoforms, in particular Na_V_1.6 and Na_V_1.7, are associated with symptoms of spontaneous (non-stimulus-induced) pain, while TTX-r isoforms contribute to cold allodynia.

### Ciguatoxin-induced gastrointestinal sensory disturbances are mediated predominantly by Na_V_1.8

In order to delineate the Na_V_ isoforms contributing to ciguatoxin-induced gastrointestinal disturbances, we established a model of P-CTX-1-induced abdominal pain. Consistent with the excitatory effect of P-CTX-1 on colonic nociceptors (Fig. 6g,h), intracolonic administration of P-CTX-1 (1 nM, 100 μl) resulted in the activation of dorsal horn neurons within the thoracolumbar spinal cord, as identified by pERK immunoreactivity (Fig. 7c). There was a significantly greater number of pERK-IR neurons following intracolonic P-CTX-1 compared to vehicle (Fig. 7d,e). The pERK-IR neurons activated by P-CTX-1 are primarily located within the superficial dorsal horn laminae I and V, indicating the activation of nociceptive processing pathways within the spinal cord. In order to evaluate behavioural changes associated with intracolonic administration of P-CTX-1, we assessed exploring and grooming behaviours using an automated rodent behavioural analysis platform (Behavioral Spectrometer, Biobserve, New Jersey, USA). Compared with administration of saline, intracolonic P-CTX-1 led to a significant increase (*p* < 0.05) in still (Fig. 7f) and grooming (Fig. 7g) behaviours as well as a significant decrease (*p* < 0.05) in orienting (Fig. 7h), rearing (Fig. 7i), walking (Fig. 7k), trotting (Fig. 7l) and running (Fig. 7m). As the selective Na_V_1.8 inhibitor A803467 blocked P-CTX-1-induced action potential firing of colonic nociceptors in our *ex vivo* preparation (Fig. 6), we tested the effect of A803467 on changes in P-CTX-1-induced behaviours. Consistent with a major role for TTX-r isoforms, specifically Na_V_1.8, in visceral pain, A803467 significantly reversed all behavioural deficits, while concomitant intracolonic administration of TTX (100 nM) did not fully reverse P-CTX-1-induced behaviours (Fig. 7f–m).

To gain further insight into the mechanisms underlying the dominant role of Na_V_1.8 in mediating the P-CTX-1-induced visceral effects, we next assessed expression of Na_V_1.6, Na_V_1.7 and Na_V_1.8 in whole thoracolumbar (TL) DRG (Fig. 7n) and found that Na_V_1.8 was the most highly expressed Na_V_ isoform, followed by Na_V_1.7 and Na_V_1.6. Single cell RT-PCR expression analysis within individual retrogradely labelled colonic DRG neurons revealed that Na_V_1.6 was expressed in 63% (15/24), Na_V_1.7 expressed in 100% (24/24) and Na_V_1.8 expressed in 88% (21/24) of thoracolumbar colonic DRG neurons (Fig. 7o). Thus, the high and nearly ubiquitous expression of Na_V_1.8 in colonic DRG neurons provides an explanation for the apparent functional selectivity of an essentially non-selective Na_V_ activator toxin like P-CTX-1.

## Discussion

Ciguatera is a circumtropical ichthyosarcotoxism linked to the consumption of fish contaminated by the ciguatoxins; heat-stable polyethers that activate neuronal Na_V_ isoforms to cause neuronal hyper-excitability. The disease is endemic in the Pacific, Caribbean and Indian Oceans and presents with predominantly gastrointestinal and sensory disturbances, muscle weakness, as well as cardiovascular effects in severe poisonings^1^. While it is established that these manifestations of ciguatoxin poisoning are likely attributable to activation of Na_V_ isoforms expressed in sensory neurons, cardiac tissue and skeletal muscle, the effects of ciguatoxin on individual Na_V_ isoforms and their relative contribution to the symptomatology of ciguatera has not been assessed systematically. Therefore, we determined the specific effects of P-CTX-1 on all Na_V_ isoforms, as well as the contribution of individual Na_V_ isoforms to action potential firing in dermal and visceral afferents. We also determined the Na_V_ isoforms contributing to ciguatoxin-induced pain of dermal and gastrointestinal origin. Overall, our results suggest that the effects of P-CTX-1 on Na_V_1.8 are particularly important in the majority of C-fibre nociceptors, while TTX-s Na_V_ isoforms, in particular Na_V_1.6 and Na_V_1.7, contribute to P-CTX-1-mediated effects on A-fibres.

P-CTX-1 is the most potent ciguatoxin congener and is thought to be responsible for the majority of symptoms in the Pacific^28^. Ingestion of fish contaminated with 0.1 ppb ciguatoxin is known to cause ciguatera, with the average pathogenic dose causing symptoms in humans estimated at 2 ng/kg^29^. In mice, intravenous dosing with 130 ng/kg P-CTX-1 was associated with peak plasma levels of approximately 60 pg/mL^30^, however unfortunately, data on plasma ciguatoxin levels in human ciguatera patients is not available.

Estimation of the concentration of the ciguatoxins in tissue is even more difficult, particularly in light of the high lipophilicity of the toxins and the corresponding large volume of distribution^31^. Based on human experiments assessing sensory effects of P-CTX-1 after intracutaneous administration, concentrations ranging from 1–10 nM are sufficient to elicit symptoms of itching, burning pain and cold allodynia^9^,^27^. Given the rapid emergence of effects after local administration, as well as the quasi-irreversible binding to Na_V_ channels, losses due to redistribution are likely minimal, suggesting that the concentrations of P-CTX-1 used for *in vitro* studies in our experiments are highly relevant to human disease.

We found that P-CTX-1 is approximately equipotent at all human Na_V_ isoforms. Although P-CTX-1 can be considered a relatively non-selective Na_V_ activator in terms of potency, we did observe significant functional differences that were Na_V_ subtype dependent.

While the voltage-dependence of activation was shifted to more hyperpolarising potentials at all Na_V_ subtypes, a hyperpolarising shift in the voltage-dependence of steady-state fast inactivation only occurred at Na_V_1.2, Na_V_1.3 and Na_V_1.9. These observed hyperpolarising shifts could lead to decreased availability of these isoforms for action potential generation and propagation *in vivo*. In addition, P-CTX-1 induced a significantly accelerated inactivation at Na_V_1.1, Na_V_1.2 and Na_V_1.3. In contrast, a significant depolarising shift in steady-state fast inactivation was observed for Na_V_1.4. Similar effects were also reported for the N440K Na_V_1.4 mutation, which is associated with paradoxical myotonia^32^, suggesting that the increase in window current effected by concomitant shifts in the voltage-dependence of activation and inactivation of Na_V_1.4 may contribute to ciguatoxin-induced muscle weakness.

P-CTX-1 also caused a small persistent current at Na_V_1.3, which may contribute to the slightly increased activity at this isoform we observed in the membrane potential assay. This activity may also explain our previous observation that P-CTX-1-induced responses in the neuroblastoma cell line SH-SY5Y are mediated predominantly through Na_V_1.3^27^. However, as Na_V_1.3 is typically expressed at high levels during development, and is reported to be essentially absent from adult rodent DRG neurons, this suggests that effects at Na_V_1.3 are unlikely to contribute substantially to the *in vivo* symptomatology of ciguatera^33^. Interestingly, a previous study reporting the emergence of a TTX-sensitive leak current after treatment with P-CTX-1 used neonatal DRGs, suggesting that this activity may have been mediated by expression of neonatal Na_V_1.3^20^.

P-CTX-1 had the most pronounced effects at Na_V_1.8, including a large shift in the voltage-dependence of activation that was particularly apparent at depolarised potentials, and a significant delay in inactivation. In contrast, P-CTX-1 did not affect the voltage-dependence of fast inactivation of Na_V_1.8. These effects are consistent with our observation of increased activation and prolonged active periods observed with single channel recordings, and the reported effects of the synthetic ciguatoxin analogue CTX3C on rat Na_V_1.8 channels, where large leak currents as well as a shift in the voltage-dependence of activation occurred, albeit at 10-fold higher concentrations and bringing into question whether the native form of CTX3C was synthesised^34^.

The binding site of the ciguatoxins and brevetoxins, all of which are believed to act at site 5 of the voltage-gated sodium channel^35^,^36^, is relatively poorly defined. Using elegant photoaffinity labelling experiments of rat brain sodium channels, the likely regions involved in binding of brevetoxin were identified as residues in domain I S6 (Thr400-Lys443; rat Na_V_1.2 numbering) and the extracellular loop following domain IV S5 (Glu1688 – Lys1735; rat Na_V_1.2 numbering)^35^.

The domain I binding site appears to be particularly important for high affinity effects of the ciguatoxins on Na_V_1.8, as chimeric channels containing domains I and II from Na_V_1.4 responded much less to synthetic ciguatoxin analogues than native Na_V_1.8, or chimeras incorporating domains I and II from Na_V_1.8^34^. Interestingly, both the domain I and domain IV regions are highly conserved across rodent and human Na_V_ isoforms, perhaps explaining at least in part the essentially non-selective effects of P-CTX-1. More subtle, subtype-specific effects on channel gating are more difficult to explain with our current knowledge of the ciguatoxin binding site. For example, the residues forming site 5 in domain I are identical between human Na_V_1.1 and Na_V_1.2, while only 2 residues (G1727 > K and D1734 > N, Na_V_1.2 numbering) differ in domain IV. It is currently unclear whether these differences could contribute to the observed effect of P-CTX-1 on channel inactivation at Na_V_1.2, but not Na_V_1.1. An alternate, perhaps more likely, explanation is that additional, as yet undefined, residues outside the immediate binding pocket contribute to subtype-specific effects on channel gating.

Adult sensory neurons predominantly express three TTX-sensitive Na_V_ isoforms Na_V_1.1, Na_V_1.6, and Na_V_1.7, as well as the TTX-resistant isoforms Na_V_1.8 and Na_V_1.9^37^,^38^. Previous studies have demonstrated that P-CTX-1 affects both TTX-sensitive and TTX-resistant Na_V_ isoforms in DRG neurons^20^,^21^. Accordingly, in the current study using cultured DRG neurons, both Na_V_1.8 and TTX-sensitive isoforms were major contributors to P-CTX-1-induced responses. However, while P-CTX-1 also affected the voltage-dependence of activation of Na_V_1.9, P-CTX-1-induced responses were unchanged in DRG neurons from Na_V_1.9^−/−^ animals. Nonetheless, some small responses remained in DRG neurons from Na_V_1.8^−/−^ animals treated with TTX, suggesting that at least a small component of Na^+^ influx induced by P-CTX-1 may be conducted by Na_V_1.9. Indeed, a small number of non-responding individual neurons were observed in cultures from Na_V_1.9^−/−^ animals. These results suggest that, at least at the concentrations assessed here, and perhaps due to CTX’s effects on voltage-dependence of fast inactivation, Na_V_1.9 is not a major contributor to P-CTX-1-mediated effects.

Experiments assessing P-CTX-1-induced action potential firing in C-fibres both from the skin-saphenous nerve and colonic preparations revealed a major role for Na_V_1.8, with the Na_V_1.8 blocker A803467 significantly reducing or blocking firing. While some dermal C-fibres were silenced by TTX, surprisingly few were affected by selective inhibition of Na_V_1.7, suggesting that in the majority of C-fibres, P-CTX-1-induced action potential firing is maintained by Na_V_1.8. This observation is consistent with previous studies reporting a contribution of Na_V_1.8 to more than 80% of the inward membrane current during the rising phase of the action potential^39^ and also a substantial decrease in C-fibre activity in response to P-CTX-1 in fibres from Na_V_1.8^−/−^ mice^9^. In terms of visceral pathways, the involvement of Na_V_1.8 in P-CTX-1 induced colonic nociceptor activation is consistent with a pronounced role for Na_V_1.8 in visceral pain, as demonstrated by Nav1.8 ^−/−^ mice displaying blunted pain and hyperalgesia to intracolonic mustard oil administration^40^.

We found that P-CTX-1-induced action potential firing in A-fibres was almost completely abolished by selective inhibition of Na_V_1.7 and substantially reduced by the Na_V_1.6 inhibitor GIIIA. These findings are consistent with the major contribution of TTX-s Na_V_ isoforms to action potential conduction in these neurons^41^,^42^. The Na_V_1.7 findings are intriguing, as Na_V_1.7 plays a key role in regulating sensory neuron excitability, owing to its unique expression pattern, as well as slow closed-state inactivation, which underpins the ability of Na_V_1.7 to generate large ramp currents in response to slow depolarisations^43^. While we could not assess expression of Na_V_1.7 in non-responding A-fibres, and distinction of Aβ and Aδ fibres based on conduction velocity is difficult in the murine skin-nerve preparation due to the limited length of the saphenous nerve, it seems plausible that nerve endings of Aδ fibres expressing Na_V_1.7 respond to P-CTX-1 with action potential firing, whereas those lacking this isoform (presumably Aβ fibres) do not. This conclusion is not only consistent with the hyperpolarising shift in the voltage-dependence of activation at Na_V_1.7 caused by P-CTX-1, which would lead to enhanced neuronal excitability and action potential firing, but also expression of Na_V_1.7 in a significant proportion of high- and low-threshold myelinated Aδ neurons but few, if any, cutaneous myelinated Aα/β low-threshold mechanosensitive units^44^. Indeed, our results showed that A-fibres responding to P-CTX-1 had slower conduction velocities than non-responders. In comparison, Na_V_1.6 is widely expressed in large diameter DRG neurons of myelinated fibres, where it is localised predominantly at the nodes of Ranvier^45^,^46^. Thus, the selective activation of some, but not all, A-fibres by P-CTX-1 suggests that the relatively minor shift in the voltage-dependence of activation at Na_V_1.6 caused by P-CTX-1 is not sufficient to induce action potential firing, and that the inhibitory actions of GIIIA likely arises from the effects on action potential propagation, which depends on Na_V_1.6 in these fibres.

We have previously shown in humans that the *in vivo* effects of P-CTX-1 after intradermal injection closely resemble the effects elicited by intraplantar injection in mice. This is evidenced by characteristic symptoms of spontaneous pain that resolve to reveal cold allodynia upon exposure to cool temperatures^9^,^27^. Whilst P-CTX-1-induced cold allodynia is mediated by TRPA1-expressing nociceptors and involves activation of Na_V_1.8, the contribution of individual Na_V_ isoforms to P-CTX-1-induced spontaneous and visceral pain have not been determined. In the current study we found that inhibition of P-CTX-1-induced acute dermal pain paralleled the pharmacology of action potential firing in A-fibres, with both selective Na_V_1.7 and Na_V_1.6 inhibition leading to a reversal of associated pain behaviours. This likely reflects the pattern of expression of Na_V_ isoforms in the sensory nerve endings of skin afferents^47^, which in turn is determined by the electrophysiological properties of individual isoforms, as well as the specific effects of P-CTX-1 on each isoform. Accordingly, in colonic sensory neurons, where Na_V_1.8 was the most highly expressed isoform, inhibition of Na_V_1.8 by A803467, a subtype-selective small molecule blocker, significantly reversed P-CTX-1-induced behavioural effects.

In terms of other Na_V_ isoforms expressed by DRG neurons, Na_V_1.1 was recently shown to mediate mechanically-evoked action potential firing in dermal and colonic sensory neurons^48^. While P-CTX-1 caused a hyperpolarising shift in the voltage-dependence of Na_V_1.1 activation, it also caused acceleration of inactivation, suggesting that the contribution of Na_V_1.1 to P-CTX-1-induced *in vivo* effects may be negligible. Indeed, we previously reported a lack of P-CTX-1-induced mechanical allodynia after intraplantar injection^9^, consistent with the lack of effect of the Na_V_1.1 inhibitor TIIIA on spontaneous pain observed in this study.

While intraperitoneal injection of P-CTX-1 gives rise to symptoms consistent with visceral hypersensitivity, including diarrhoea, writhing and abdominal contractions, potentially confounding effects on global nerve conduction velocity make it poorly suited to delineating the pharmacology of ciguatoxin-induced visceral pain^49^,^50^. In contrast, our model based on intracolonic administration of P-CTX-1, which caused activation of colonic nociceptors and activation of nociceptive processing regions in the spinal cord, led to more subtle effects on behaviour, including decreased locomotion and exploring behaviours. However, while systemic dosing with the Na_V_1.8 inhibitor A803467 led to a substantial decrease in P-CTX-1-induced behavioural abnormalities, the TTX-sensitive Na_V_ isoforms contributing to the *in vivo* effects of ciguatoxin remain to be determined. A likely candidate is Na_V_1.7, albeit Na_V_1.1 was recently shown to contribute to visceral mechanical hypersensitivity in models of irritable bowel syndrome^48^. A particularly interesting aspect that remains to be addressed is whether oral administration of subtype-selective peptidic Na_V_ inhibitors could ameliorate the gastrointestinal symptoms associated with ciguatera.

Finally, while this study focused on the subtype-selective effects of P-CTX-1 on Na_V_ isoforms, the ciguatoxins are also known to inhibit K^+^ channel function. However, while effects on both delayed rectifier and A-type channels have been reported^51^, the pharmacology of P-CTX-1 at heterologously expressed K^+^ channel isoforms has not been systematically assessed. Accordingly, the contribution of this alternate pharmacology to the symptomatology of ciguatera remains to be determined, particularly since the subtle subtype-specific effects of Na_V_ gating are unlikely to fully account for the pathognomonic symptom of cold allodynia.

In conclusion, we have shown that in terms of potency P-CTX-1 is a relatively non-selective activator of human Na_V_ subtypes that shifts the voltage-dependence of activation to more hyperpolarising potentials for all Na_V_ subtypes. However, P-CTX-1 induces distinct functional effects on Na_V_ subtypes, including increasing the inactivation time constant at Na_V_1.8, increasing the slope factor of the conductance-voltage curve at Na_V_1.7, and increasing the peak current at Na_V_1.6. Correspondingly, the *in vivo* effects induced by P-CTX-1 appear to be driven predominantly by Na_V_1.6, Na_V_1.7 and Na_V_1.8. Thus, we conclude that the behavioural effects associated with ciguatera reflect the expression pattern of Na_V_ isoforms in peripheral nerve endings, as well as their CTX-induced contribution to membrane depolarisation, action potential initiation and propagation. Accordingly, selective inhibitors of Na_V_1.7 and Na_V_1.8, and to a lesser extent Na_V_1.6 which is also found in motor nerves, may be promising therapeutics for management of the symptomatology of ciguatera in the clinic.

## Materials and Methods

### Ethics and animals

Ethical approval for experiments involving animals was obtained from the local animal ethics committees at the University of Queensland, the University of Adelaide, the South Australian Health and Medical Research Institute (SAHMRI) and the University of Erlangen in accordance with local legislation. Experiments involving animals were conducted in accordance with the Animal Care and Protection Act Qld (2002), the Australian Code of Practice for the Care and Use of Animals for Scientific Purposes, 8^th^ edition (2013) and the International Association for the Study of Pain Guidelines for the Use of Animals in Research. Adult male C57BL/6, Na_V_1.8^−/−^ and Na_V_1.9^−/−^ mice (John N. Wood, University College London, London, UK; congenic to C57BL/6 or backcrossed for at least 6 generations on the C57BL/6 background, respectively) and their age-matched litter controls were used. All animals were genotyped using previously reported primers^52^,^53^.

Animals were randomly assigned to treatment groups using a stratified randomisation strategy and all assessors were blinded to genotype or treatment. General health and wellbeing was monitored daily and no animals were withdrawn from the study. Sample sizes of each experiment are detailed in the figure legends of the corresponding figure.

### Chemicals and drugs

P-CTX-1 was isolated and purified from moray eel viscera to >95% purity as previously described by Lewis *et al*.^28^ and stored as a concentrated stock in 50% methanol/50% H_2_O. P-CTX-1 was diluted in relevant assay buffers in the presence of 0.1% bovine serum albumin Cohn Fraction V (BSA) (BioScientific, Kirrawee, NSW, Australia) to avoid losses to plastic surfaces. Veratridine was obtained from Ascent Scientific (Bristol, United Kingdom). All other reagents were obtained from Sigma Aldrich (Castle Hill, NSW, Australia) unless otherwise specified.

### Cell Lines and culture

Stable human embryonic kidney cells (HEK293) constitutively expressing human Na_V_ isoforms Na_V_1.1–1.8 (SB Biomedical, Glasgow, United Kingdom). These cells were routinely maintained in minimal essential medium (MEM) (Sigma-Aldrich, Australia) supplemented with 10% foetal bovine serum (FBS) (Bovogen Biologicals, France), 2 mM L-glutamine and selection antibiotics as recommended by the manufacturer. For electrophysiological studies, CHO cells stably expressing hNa_V_1.8 in a tetracycline-inducible system (Chantest, OH, USA) were cultured in Ham’s F-12 containing 10% v/v foetal bovine serum and selection antibiotics as recommended by the manufacturer. To induce hNa_V_1.8 expression, cells were cultured in the presence of tetracycline (1 μg/mL) for 24 h at 27 °C. All cell lines were grown in a humidified 5% CO_2_ incubator at 37 °C, grown to 70–80% confluence, and passaged every 3–4 days using TrypLE Express (Invitrogen). HEK293 stably expressing hNa_V_1.9 were generated and maintained by Icagen Inc. (Durham, NC, USA) as described^54^.

### FLIPR^Tetra^ Membrane Potential Assay

Activity of P-CTX-1 and Pn3a at hNa_v_1.1–1.8 was assessed using a fluorescent plate reader (FLIPR^Tetra^) membrane potential assay as previously described^55^. In brief, cell lines (HEK293 Na_v_1.1–1.8) were plated on 384-well black-walled imaging plates (Corning) at a density of 10 000–15 0000 cells per well 48 hours before loading with 20 μL of red membrane potential dye (proprietary formulation) (Molecular Devices, Sunnyvale, CA). Cells were incubated with the membrane potential dye for 30 min at 37 °C before the addition of P-CTX-1 and veratridine or deltamethrin by the FLIPR^tetra^ system. After the first addition (P-CTX-1), fluorescence was measured (excitation 515–545 nm; emission: 565–625 nm) for a period of 5 min to determine the effects of P-CTX-1 alone. Following this 5 min exposure, veratridine (5–20 μM) or deltamethrin (100 μM) was added and fluorescence was measured for a further 5 min. Data was recorded and converted to response over baseline using Screenworks 3.2.0.14. For concentration-response curves, maximum values from the response after addition of agonist were plotted against agonist concentration and a 4-parameter logistic Hill equation was fitted to the data using GraphPad Prism Version 6.00 (San Diego, CA).

### Electrophysiology

#### Electrophysiology (Na_V_1.1–1.8)

For Na_V_1.1–1.8, whole-cell patch-clamp experiments were performed on a QPatch-16 automated electrophysiology platform (Sophion Bioscience, Ballerup, Denmark) using 16-channel planar patch chip plates (QPlates; Sophion Bioscience) with a patch hole diameter of 1 μm and resistance of 2 ± 0.02 MΩ. Cell positioning and sealing parameters were set as follows: positioning pressure −60 mbar, minimum seal resistance 0.1 GΩ, holding potential −100 mV, holding pressure −20 mbar. Whole-cell currents were filtered at 5 kHz and acquired at 25 kHz.

HEK-293 cells expressing hNa_V_1.1–1.7 or tetracycline inducible CHO cells expressing hNa_V_1.8 (as Na_V_1.8-HEK cells are unsuitable for patch clamping) were harvested at 70–80% confluence, by washing with Ca^2+^ and Mg^2+^ free Dulbecco’s phosphate-buffered saline and incubating with Detachin (Bio-Scientific, NSW, Australia) at 37 °C for 2–5 min. Dissociated cells were then resuspended in Ex-Cell ACF CHO Medium with 25 mM HEPES (Sigma-Aldrich, NSW, Australia) and transferred to the QStirrer (Sophion) and allowed to recover for 30 min. The extracellular solution contained in mM: NaCl 145, KCl 4, CaCl_2_ 2, MgCl_2_ 1, HEPES 10 and glucose 10. The pH was adjusted to 7.4 with NaOH and osmolarity was adjusted with sucrose to 305 mOsm. The intracellular solution contained in mM: CsF 140, EGTA/CsOH 1/5, HEPES 10 and NaCl 10. The pH was adjusted to 7.3 with CsOH and osmolarity adjusted with sucrose to 320 mOsm. P-CTX-1 was diluted in extracellular solution with 0.1% BSA to a concentration of 10 nM and incubated for 5 min. All P-CTX-1 effects were compared to pre-toxin control parameters within the same cell.

Current (I)–voltage (V) relationships were obtained with a holding potential of −80 mV followed by a pre-pulse of −100 mV for 50 ms and a series of 50 ms step pulses that ranged from −80 to + 60 mV in 5 mV increments before returning to a holding potential of −80 mV (repetition interval 5 s). Conductance-voltage relationships were obtained by calculating the conductance (G) at each voltage (V) using the equation *G* = *I*/(*V* − *V*_*rev*_), *w*here *V*_*rev*_ is the reversal potential. Conductance-voltage curves were fitted with a Boltzmann equation: G_Na_ = G_Na,max_/1 + exp [(V_m_ − V_1/2_)/*k*], where G_Na_ is the voltage-dependent sodium conductance, G_Na,max_ is the maximal sodium conductance, V_1/2_ is the potential at which activation is half-maximal, V_m_ is the membrane potential, and *k* is the slope factor.

Voltage dependence of steady-state fast inactivation was measured using a series of 500 ms pre-pulses, ranging from −120 to −10 mV in 10 mV increments, followed by a 20 ms pulse of −20 mV for Na_V_1.1–1.7 and +10 mV for Na_V_1.8 to assess the available non-inactivated channels (repetition interval 30 s). Peak inward currents (I) were normalised to the maximal inward current (I_max_) and fitted using a Boltzmann equation: I/I_max_ = 1/(1 + exp [(V_m_ − V_1/2_)/*k*)], where I_max_ is the maximal inward current, V_1/2_ is the half-maximal sodium current, V_m_ is the pre-conditioning pulse potential, and *k* is the slope factor.

Time to peak, fast inactivation time constants and persistent current were determined by a single-pulse protocol, using a holding potential of −80 mV, followed by a pre-pulse of −100 mV for 50 ms and then a 50 ms pulse of −20 mV for Na_V_1.1–1.7 and +10 mV for Na_V_1.8. Time to peak was measured from pulse onset to maximal current, fast inactivation time constants were calculated by fitting current decay traces with a single exponential function, and persistent current was determined as the mean current between 40 and 50 ms after pulse onset.

#### Electrophysiology (Na_V_1.9)

For hNa_V_1.9, whole-cell patch-clamp experiments were performed by Icagen Inc (Durham, NC, USA) on a PatchXpress automated electrophysiology platform (Molecular Devices, Sunnyvale, CA, USA) using HEK-293 cells stably expressing hNa_V_1.9. The extracellular solution for these studies contained in mM: NaCl 135, KCl 5.4, CaCl_2_ 2, MgCl_2_ 1, HEPES 10 and glucose 5. The pH was adjusted to 7.4 with NaOH with a measured osmolarity of 300 mOsm. The intracellular solution contained in mM: CsF 135, EGTA 5, CsCl 10, HEPES 10 and NaCl 5. The pH was adjusted to 7.4 with CsOH with a measured osmolarity of 298 mOsm. P-CTX-1 (1 nM) was diluted in extracellular solution with 0.1% BSA to a concentration of 1 nM and incubated for 5 min. All P-CTX-1 effects were compared to pre-toxin control parameters within the same cell. Current (I)–voltage (V) curves were obtained with a holding potential of −140 mV, then a series of 100 ms step pulses that ranged from −140 to 0 mV in 10 mV increments, followed by a 20 ms pulse of −40 mV to assess the available non-inactivated channels (repetition interval 10 s). Conductance-voltage curves and voltage dependence of steady-state fast inactivation curves were then calculated and fitted as described above.

#### Single Channel Recordings

Single channel recordings were done in the excised, outside-out patch configuration at room temperature (22 ± 1 °C). Currents were elicited by stepping the clamped voltage from −100 mV to −20 mV before and after exposing the recorded path to 1 nM P-CTX-1 for 10–12 min. The intracellular solution was composed of (in mM): 145 CsCl, 2 CaCl_2_, 2 MgCl_2_, 10 HEPES, and 5 EGTA, adjusted to pH 7.4 with CsOH. The control extracellular solution was composed of (in mM): 140 NaCl, 5 KCl, 2 CaCl_2_, 1 MgCl_2_, 10 HEPES, and 10 D-glucose, adjusted to pH 7.4 with NaOH. Bovine serum albumin at 0.01% was added to all working extracellular solutions.

Recording electrodes were fire polished and had resistances of 5–9 MΩ when filled with intracellular solution. Single channel currents were recorded using an Axon 200B amplifier (Molecular Devices), filtered (−3 dB, 4-pole Bessel) at 5 kHz and sampled at 20 kHz. Currents were filtered offline to 2 kHz for making figures. The single channel active period durations were estimated using QuB software, from idealising events longer that 70 μs (dead time). Single channels activations were defined by a critical shut duration (t_crit_) of 5–10 ms (large amplitude) and 20–70 ms (small amplitude). t_crit_ was calculated for each patch by equalising the area under the longest two shut components in the shut dwell histograms. Data are presented as mean ± SEM from 3–5 patches in all recording conditions.

### Retrograde tracing of colonic DRG neurons

Cholera toxin subunit B conjugated to AlexaFluor 488 (CTB-488; Invitrogen, Carlsbad, CA) was injected at three sub-serosal sites within the wall of the distal colon of C57BL/6 mice. After 4 days, animals were humanely killed by CO_2_ inhalation for subsequent DRG removal and culture^56^,^57^.

### Dorsal root ganglion isolation and culture

Dorsal root ganglion neurons were isolated and cultured as previously described^9^,^25^,^48^,^58^. For Na^+^ imaging experiments, DRGs from T1-L6 were isolated from adult male C57/BL6 mice, male Na_V_1.8^−/−^ mice or male Na_V_1.9^−/−^ mice euthanised by CO_2_ inhalation and collected in DMEM supplemented with 50 μg/ml gentamicin (Sigma), 100 U/ml penicillin, 100 ug/ml streptomycin and 0.25 μg/ml amphotericin B (Life Technologies). DRGs were then incubated for 30 min at 37 °C and 5% CO_2_ in dissociation media containing 1 mg/ml collagenase (Sigma) and 0.1 mg/ml protease (Sigma). After three wash steps, cells were triturated through a flame-polished glass Pasteur pipette and cultured for 24 h in TNB 100 solution supplemented by TNB 100 lipid-protein-complex, 1 nM NGF, 100 μg/ml streptomycin and penicillin (all from Biochrom, Berlin, Germany) and 200 μg/ml glutamine (Life Technologies) or Neurobasal medium supplemented with B27, 1 nM NGF and 100 μM glutamine (all from Life Technologies, Mulgrave, Vic, Australia) for high content imaging.

For Ca^2+^ imaging experiments, T10-L1 DRGs from retrogradely traced mice were surgically removed and were digested with 4 mg/mL collagenase II (GIBCO, Life Technologies) plus 4 mg/mL dispase (GIBCO) for 30 min at 37 °C, followed by 4 mg/mL collagenase II for 10 min at 37 °C. Neurons were then mechanically dissociated into a single-cell suspension via trituration through fire-polished Pasteur pipettes. Neurons were resuspended in DMEM (GIBCO) containing 10% FCS (Invitrogen), 2 mM L-glutamine (GIBCO), 100 μM MEM non-essential amino acids (GIBCO), 100 mg/ml penicillin/streptomycin (Invitrogen) and 100 ng/ml NGF (Sigma). Neurons were spot-plated on coverslips coated with poly-D-lysine (800 μg/ml) and laminin (20 μg/ml) and maintained at 37 °C in 5% CO_2_. Comparisons were made between retrogradely labeled colonic DRG neurons and non-labelled DRG neurons.

### Na^+^ and Ca^2+^ imaging of cultured DRG neurons

To assess the effect of P-CTX-1 (1 nM) on Na^+^ and Ca^2+^ influx in cultured DRG neurons, cells were plated on PDL-coated glass coverslips and after 24 hours in culture incubated with 10 μM Sodium Green-AM (Na^+^ indicator) or 2.5 μM Fura2-AM (Ca^2+^ indicator) respectively. Sodium Green -AM was incubated for 1 h at 37 °C while Fura2-AM was incubated for 30 min at room temperature in extracellular fluid (ECS, composition: NaCl 145 mM, KCl 5 mM, CaCl2 1.25 mM, MgCl2 1 mM, glucose 10 mM, HEPES 10 mM) containing 0.02% (v/v) pluronic acid in DMSO. After a brief wash, coverslips were transferrerd to the recording chamber and Na^+^ or Ca^2+^ responses were measured at room temperature (~22 °C). Sodium Green was excited at 507 nm with a Polychrome V monochromator (Till Photonics). Images were exposed for 200 μs and acquired at a rate of 1 Hz with a peltier-cooled slow-scan CCD camera system (Imago Sensicam QE, Till Photonics). Data were recorded and further analysed using TILLvisION 4.0.1.3 software (Till Photonics). After a baseline read with extracellular solution or TTX (300 nM), DRG neurons were stimulated with 1 nM P-CTX-1, followed by a final stimulation with 30 mM KCl to identify viable neurons. Na^+^ responses were analysed as the total fluorescence with responses >1000 fluorescence units defined as responders.

Fura-2 was excited at 340 and 380 nm using a Nikon TE300 Eclipse microscope equipped with a Sutter DG-4/OF wavelength switcher, Omega XF04 filter set for Fura-2, Photonic Science ISIS-3 intensified CCD camera, and Universal Interface Card MetaFluor software. Fluorescence images were obtained every 20 s using a 20x objective. Data were recorded and further analysed using Xcellence software (Olympus) after a baseline fluorescence read with extracellular solution or in the presence of TTX (500 nM). Colonic DRG neurons (identified by the presence of the 488 tracer) were stimulated with 1 nM P-CTX-1, followed by a final stimulation with 40 mM KCl to identify viable neurons. Ca^2+^ responses were presented as the fluorescence ratio 340/380. Ca^2+^ maximum peak responses elicited by P-CTX-1 were calculated by subtracting maximum Fura-2 fluorescence (340/380) values to the Fura-2 fluorescence (340/380) values before P-CTX-1 addition.

### Single fibre recordings using the murine skin-saphenous nerve preparation

To characterise the pharmacology of P-CTX-1-induced effects on C- and A-fibre afferents, we used extracellular recordings from the murine skin-saphenous nerve preparation as previously described^59^. The hairy skin of the dorsal hind paw and lower leg of male adult C57BL/6 mice was carefully dissected together with the saphenous nerve, placed in an organ bath chamber with the epithelial side secured in a downward position using white soft paraffin and continuously perfused with carbogenated SIF (composition (mM): NaCl (107.8), KCl (3.5), MgSO_4_ (0.69), NaHCO_3_ (26.2), NaH_2_PO_4_ (1.67), Na-gluconate (9.64), glucose (5.55), sucrose (7.6) and CaCl_2_ (1.53); pH 7.3). The desheathed saphenous nerve was placed on a mirror in an adjacent paraffin oil-filled recording chamber and teased into filaments until recordings from single, mechanically excitable receptive fields was achieved. Fibres were classified according to conduction velocity (C-fibre < 1 m/s, A-fibre 1.6–12 m/s) after electrical stimulation of the receptive field with a bipolar teflon coated steel microelectrode (impedance ~ 1 MΩ). The dermal receptive field was then isolated using a plastic ring, and after a 5 min recording period to determine baseline activity, P-CTX-1 (0.5 nM) was perfused at a rate of 5 mL/min at 32 °C for up to 30 min. Fibres without an increase in spontaneous action potential discharge rate after 30 min were considered non-responders. For responding fibres, once spontaneous action potential firing was stable for at least 5 min, Na_V_ subtype-selective inhibitors were sequentially added every 15 min to the perfusate to achieve final concentrations of the Na_V_1.7 inhibitor Pn3a (1 μM), the Na_V_1.6 inhibitor GIIIA (10 μM), TTX (1 μM) and the Na_V_1.8 inhibitor A803467 (10 μM). Data was recorded and analysed using DAPSYS (version 8).

### Single fibre colonic afferent recordings using the murine colon-splanchnic nerve preparation

C57BL/6 male mice were humanely killed by CO_2_ inhalation. The colon and rectum with attached mesentery and splanchnic nerves were removed and afferent recordings performed as described previously^24^,^25^,^60^. Briefly, the colon was removed and pinned flat, mucosal side up, in a specialised organ bath. The colonic compartment was superfused with a modified Krebs solution (in mM: 117.9 NaCl, 4.7 KCl, 25 NaHCO_3_, 1.3 NaH_2_PO_4_, 1.2 MgSO_4_, 2.5 CaCl_2_, 11.1 D-glucose), bubbled with carbogen (95% O_2_, 5% CO_2_) at a temperature of 34 °C. All preparations contained the L-type calcium channel antagonist nifedipine (1 μM) to suppress smooth muscle activity and the prostaglandin synthesis inhibitor indomethacin (3 μM) to suppress potential inhibitory actions of endogenous prostaglandins. The nerve bundle was extended into a paraffin-filled recording compartment in which finely dissected strands were laid onto a mirror, and single fibre placed on the platinum recording electrode. Action potentials generated by mechanical or chemical stimuli to the colon’s receptive field, pass through the fibres into a differential amplifier, filtered, sampled (20 kHz) using a 1401 interface (Cambridge Electronic Design, Cambridge, UK) and stored on a PC for off-line analysis. P-CTX-1 (1 nM) was added for 5 min via a small metal ring placed over the receptive field of interest in selective experiments the Na_V_1.8 channel inhibitor A803467 (10 μM) was pre-incubated for 10 min prior co-incubation with P-CTX-1. This route of administration has been previously shown to reproducibly activate afferents^25^,^48^,^61^. Action potentials were analysed off-line using the Spike 2 wavemark function and discriminated as single units on the basis of distinguishable waveform, amplitude and duration.

### Colonic afferent classification

Receptive fields were identified by systematically stroking the mucosal surface with a still brush to activate all subtypes of mechanoreceptors. Categorisation of afferents properties was in accordance with our previously published classification system^59^,^62^. Briefly, splanchnic nociceptors have high-mechanical activation thresholds, responding to noxious distension (40 mm Hg), stretch (≥7 g) or von Frey hair filaments (2 g) but do not respond to fine mucosal stroking (10 mg von Frey hairs; vfh).

### Animal model of P-CTX-1-evoked nocifensive behaviours

To assess the pharmacology of P-CTX-1-evoked nocifensive behaviours, a single dose of P-CTX-1 diluted in sterile saline was administered by subcutaneous injection to the hind paw (intraplantar, i.pl., 40 μl, 10 nM) under brief isoflurane (3%) anaesthesia. To assess the effect of Pn3a on Na_V_1.7-mediated responses, a single dose of the selective Na_V_1.7 activator OD1 (40 μl, 300 nM) was administered by i.pl. injection under brief isoflurane (3%) anaesthesia together with increasing concentrations of Pn3a (Suppl. Fig. 1). Nocifensive behaviours including paw lifting, licking and flinching were rated by a blinded observer over a 5 min period. To assess the effects of pharmacological modulators on the development of P-CTX-1 induced pain, compounds were either administered as a second intraplantar injection (TTX, 2 μM) or co-administered with P-CTX-1 by i.pl. injection as appropriately concentrated solutions (TTX, 1 μM; A803467, 10 μM; Pn3a 1 μM; GIIIA, 10 μM). To assess cold allodynia in ciguatoxin-treated animals, mice were placed on a temperature-controlled Peltier plate (Ugo Basile, Comerio, Italy) once spontaneous pain behaviours subsided to <10 counts/5 min and the number of paw shakes, lifts, licks or flinches was quantified over a 5 min period at 15 °C. Injection of equal volumes of sterile saline did not elicit any nocifensive behaviour.

### Animal model of P-CTX-1-evoked visceral pain

To assess the contribution of Na_V_ isoforms to the gastrointestinal symptoms associated with ciguatera, we established a novel model of P-CTX-1-induced visceral hypersensitivity. Male C57BL/6 mice house on AlphaDri bedding were fasted overnight with ad-libitum access to water. P-CTX-1 (10 nM, 50 μl) in sterile saline was administered under light isoflurane (3%) anaesthesia via a soft, flexible, round-tipped catheter that was advanced 3 cm from the anus. The Na_V_1.8 blocker A-803467 (100 mg/kg) was diluted in PEG400 and administered in a volume of 2 μl/g by intraperitoneal injection as previously described^62^. To avoid systemic adverse effects TTX (100 nM) was co-administered by intra-colonic administration with P-CTX-1. Animals were allowed to recover for 15 min and then placed into the recording chamber of a Behavioral Spectrometer (Behavioral Instruments, Hillsborough, NJ, USA) for automatic detection of still, grooming, orienting, rearing and locomotion behaviours as previously described by combining video and vibration analysis^64^.

### Phosphorylated MAP kinase ERK 1/2 (pERK) quantification in spinal cord

Mice were anaesthetised in an isofluorane chamber and received an enema of either vehicle (0.3% bovine serum albumin in 0.1 M phosphate buffer saline) or P-CTX-1 (1 nM in 0.3% bovine serum albumin in 0.1 M phosphate buffer saline). After 5 minutes mice were injected with an anaesthetic overdose (0.125 ml/250 g sodium pentobarbitone) and within 4 min underwent transcardial perfuse fixation with warm saline (0.85% physiological sterile saline) followed by ice-cold 4% paraformaldehyde in 0.1 M phosphate buffer (Sigma-Aldrich, MO, USA). Following transcardial perfusion thoracolumbar (T10-L1) spinal cord was removed, using the lowest rib as an anatomical marker of T13, and post-fixed for 16 hours at 4 °C in 4% paraformaldehyde in 0.1 M phosphate buffer. Following fixation spinal cord were cryoprotected in 30% sucrose/phosphate buffer (Sigma-Aldrich, MO, USA) overnight at 4 °C and then placed in 50% Optimal Cutting Temperature compound (OCT; Tissue-Tek, Sakura Finetek, CA, USA) in 30% sucrose/phosphate buffer solution for 7 h, before block freezing in 100% OCT. Frozen sections (15 μm) were cut using a cryostat and placed onto saline coated slides for fluorescent immunohistochemistry.

Immunohistochemistry for pERK was performed in a paired fashion, with tissue from saline or P-CTX-1 treated mice run simultaneously. Following 20 min of air drying, sections were flushed 3 times with PBS and incubated with 5% normal chicken serum/0.2% Triton-X 200 (Sigma-Aldrich, MO, USA) in PBS (0.2% TX-PBS) for 30 min at room temperature to block non-specific binding of secondary antibodies. Sections were then incubated for 18 h at room temperature with monoclonal anti-sera rabbit anti-phospho-p44/42 MAPK (Erk1/2) (Thr202/Tyr204) (pERK; 1:200; Cell Signalling Technology #4370) diluted in 0.2% TX-PBS. Sections were then washed 3 times with 0.2% TX-PBS before being incubated for 1 h at room temperature with secondary antibody chicken anti-rabbit AlexaFluorR488 (AF-488). Negative controls were prepared as above with the primary antibody omitted.

### Na_V_ isoform expression studies using Quantitative-Reverse-Transcription-Polymerase-Chain-Reaction (QRT-PCR)

#### Whole DRG QRT-PCR

RNA was extracted isolated using the PureLink RNA Micro kit (Invitrogen, cat #12183-016) and DNAse treated (LifeTechnologies cat #12185-010) following the manufacturer’s instruction. RNA was stored at −80 °C in aliquots of 1–3 μl. QRT-PCR was performed using EXPRESS One-Step Superscript^®^ qRT-PCR Kit (Life Technologies cat #11781-01 K) with commercially available hydrolysis probes (TaqMan^®^; LifeTechnologies, see Table 3 for details) and DEPC-treated water (AMBION cat #AM9916). For each reaction, 10 μl qPCR SuperMix, 1 μl TaqMan^®^ primer assay, 0.04 μl ROX, 2 μl water, 2 μl SuperScript Reverse Transcriptase and 5 μl RNA (45 ng/well, except human colon biopsies at 30 ng/well) was used in all experiments. Beta-actin was used as reference gene. PCR runs were performed in duplicate for each sample on a 7500 Fast machine (Applied Biosystems).

#### Single Cell PCR of colonic DRG neurons

AlexaFluor488 conjugated cholera-toxin subunit β retrograde traced single cells were picked using a micromanipulator under an Olympus microscope equipped with appropriate fluorescent filter. Cells were under a continuous flow of sterile and RNA/DNAse free PBS in order to reduce contamination. After a cell was picked, the glass capillary was broken into a tube containing 10 μl of lysis buffer and DNAse (TaqMan^®^ Gene Expression Cells-to-CT™ Kit). For every coverslip a bath control was analysed together with other samples. Tubb3 expression served as positive control, with GFAP expression measured to exclude contamination with glial cells.

### Statistical analysis

Data are represented as mean ± SEM. Statistical significance was defined as *p* < 0.05 and was determined using paired or unpaired student’s t-tests and One-way ANOVA analysis with post-tests as indicated. Statistical analysis was performed using GraphPad Prism Version 6.00.

## Additional Information

**How to cite this article:** Inserra, M. C. *et al*. Multiple sodium channel isoforms mediate the pathological effects of Pacific ciguatoxin-1. *Sci. Rep.* **7**, 42810; doi: 10.1038/srep42810 (2017).

**Publisher's note:** Springer Nature remains neutral with regard to jurisdictional claims in published maps and institutional affiliations.

## Supplementary Material

Supplementary Information

## Figures and Tables

**Table 1:** Effects of P-CTX-1 on Na_V_ channel voltage dependence of activation and steady-state fast inactivation.

	P-CTX-1	Maximal Peak Current (I/Imax)	Activation		Inactivation	
			V_1/2_ (mV)	*k* (mV)	V_1/2_ (mV)	*k* (mV)
Na_V_1.1	—	0.99 ± 0.00	−20.2 ± 0.4	4.5 ± 0.3	−51.8 ± 1.2	−6.7 ± 1.1
	+	0.95 ± 0.04	−25.3 ± 0.7*	4.7 ± 0.6	−53.8 ± 1.3	−6.9 ± 1.2
Na_V_1.2	—	1.00 ± 0.00	−20.6 ± 0.3	4.4 ± 0.3	−53.4 ± 0.6	−4.1 ± 0.5
	+	0.86 ± 0.09	−26.9 ± 0.8*	4.9 ± 0.7	−60.3 ± 1.2*	−5.6 ± 1.1*
Na_V_1.3	—	1.00 ± 0.00	−19.7 ± 0.3	4.9 ± 0.2	−58.0 ± 0.5	−4.1 ± 0.5
	+	1.08 ± 0.04	−24.7 ± 0.3*	3.9 ± 0.2*	−60.1 ± 0.6*	−4.2 ± 0.7
Na_V_1.4	—	0.93 ± 0.04	−36.8 ± 0.4	1.8 ± 0.3	−63.8 ± 0.5	−4.6 ± 0.4
	+	0.74 ± 0.16	−40.2 ± 0.8*	2.8 ± 0.7	−57.3 ± 1.5*	−5.8 ± 1.4
Na_V_1.5	—	0.93 ± 0.03	−37.6 ± 1.0	4.6 ± 0.9	−72.3 ± 0.6	−5.8 ± 0.6
	+	0.85 ± 0.07	−43.7 ± 1.3*	4.8 ± 1.1	−72.8 ± 0.8	−6.2 ± 0.7
Na_V_1.6	—	1.00 ± 0.00	−19.2 ± 0.2	4.8 ± 0.2	−56.0 ± 0.4	−5.1 ± 0.3
	+	1.11 ± 0.03*	−22.6 ± 0.2*	3.5 ± 0.2*	−57.1 ± 0.9	−5.4 ± 0.8
Na_V_1.7	—	0.91 ± 0.02	−33.2 ± 0.6	3.2 ± 0.5	−62.4 ± 0.4	−4.4 ± 0.4
	+	0.74 ± 0.10	−38.9 ± 1.5*	6.4 ± 1.4*	−64.4 ± 0.9	−5.7 ± 0.8
Na_V_1.8	—	0.94 ± 0.02	−5.6 ± 0.6	7.7 ± 0.6	−35.3 ± 1.7	−8.7 ± 1.5
	+	1.00 ± 0.08	−16.9 ± 0.3*	4.0 ± 0.2*	−36.1 ± 1.7	−7.9 ± 1.5
Na_V_1.9	—	0.99 ± 0.01	−48.3 ± 0.7	7.8 ± 0.6	−31.1 ± 2.1	−11.4 ± 1.6
	+	1.06 ± 0.15	−57.2 ± 1.1*	8.4 ± 1.0	−41.7 ± 2.6*	−12.7 ± 2.2

Electrophysiological parameters at hNa_V_1.1–hNa_V_1.9 heterologously expressed in HEK293 cells were assessed using whole-cell voltage-clamp recordings. Data are reported as mean ± SEM from 4–10 cells. *Indicates P < 0.05 compared to control.

**Table 2:** Effects of P-CTX-1 on Na_V_ channel activation and inactivation kinetics.

	P-CTX-1	Time to peak (ms)	Inactivation Time Constant (ms)
Na_V_1.1	—	1.23 ± 0.05	2.10 ± 0.26
	+	1.15 ± 0.10	1.20 ± 0.17*
Na_V_1.2	—	1.06 ± 0.05	1.45 ± 0.17
	+	0.78 ± 0.07*	0.79 ± 0.04*
Na_V_1.3	—	1.08 ± 0.09	0.89 ± 0.09
	+	1.05 ± 0.07	0.61 ± 0.05*
Na_V_1.4	—	0.69 ± 0.04	0.35 ± 0.04
	+	0.88 ± 0.11	0.46 ± 0.04
Na_V_1.5	—	0.86 ± 0.06	0.85 ± 0.11
	+	0.81 ± 0.07	1.00 ± 0.18
Na_V_1.6	—	1.03 ± 0.09	1.38 ± 0.22
	+	1.03 ± 0.08	1.09 ± 0.18
Na_V_1.7	—	1.09 ± 0.09	0.47 ± 0.04
	+	0.91 ± 0.06	0.49 ± 0.04
Na_V_1.8	—	1.06 ± 0.07	1.51 ± 0.21
	+	1.32 ± 0.10	3.06 ± 0.43*

Electrophysiological parameters at hNa_V_1.1-hNa_V_1.9 heterologously expressed in HEK293 cells were assessed using whole-cell voltage-clamp recordings. Data are reported as mean ± SEM from 4–10 cells. *Indicates P < 0.05 compared to control.

**Table 3:** TaqMan^®^ hydrolysis probes used for quantitative PCR.

Target	Target (official* gene symbol)	*Aliases*	Product code	Product size (bp)	NCBI Ref Seq
sodium channel, voltage-gated, type VIII, alpha	*Scn8a*	*Na*_*V*_*1.6*	Mm00488110_m1	68	NM_001077499.2; NM_011323.3
sodium channel, voltage-gated, type IX, alpha	*Scn9a*	*Na*_*V*_*1.7*	Mm00450762_s1	74	NM_001290674.1; NM_001290675.1
sodium channel, voltage-gated, type X, alpha	*Scn10a*	*Na*_*V*_*1.8*	Mm00501467_m1	82	NM_001205321.1; NM_009134.3
