# Supplementary material for: Multiple sodium channel isoforms mediate the pathological effects of Pacific ciguatoxin-1
Source: Sci Rep. 2017 Feb 22;7:42810. doi: 10.1038/srep42810 (PMC5320492; doi:10.1038/srep42810)
Supplement: Supplementary Information [file srep42810-s1.pdf]

Supplementary information

Multiple sodium channel isoforms mediate the pathological effects of Pacific ciguatoxin-1

Marco C. Inserra[1,#], Mathilde R. Israel[1,#], Ashlee Caldwell[2,3,#], Joel Castro[2,3,#], Jennifer R. Deuis[1,#], Andrea M. Harrington[2,3], Angelo Keramidas[4], Sonia Garcia-Caraballo[2,3], Jessica Maddern[2,3], Andelain Erickson[2,3], Luke Grundy[2,3], Grigori Y. Rychkov[3], Katharina Zimmermann[5], Richard J. Lewis[1], Stuart M. Brierley[2,3,*], Irina Vetter[1,6*]

# contributed equally to this work
* corresponding authors
stuart.brierley@flinders.edu.au
i.vetter@uq.edu.au

Running title: (40 characters) Ciguatoxin Na^+ channel subtype-selectivity
Keywords: (5) Ciguatoxin/Visceral/Pain/Sodium channel/Selectivity/ciguatera

[1] Centre for Pain Research, Institute for Molecular Bioscience, The University of Queensland, 306 Carmody Rd, St Lucia, Queensland 4072, Australia.

[2] Visceral Pain Group, South Australian Health and Medical Research Institute (SAHMRI), School of Medicine, Flinders University, Adelaide, South Australia 5000, Australia.

[3] Centre for Nutrition and Gastrointestinal Diseases, Discipline of Medicine, University of Adelaide, South Australian Health and Medical Research Institute (SAHMRI), North Terrace, Adelaide, South Australia 5000, Australia.

[4] Queensland Brain Institute, The University of Queensland, St Lucia, Queensland 4072, Australia.

[5] Klinik für Anästhesiologie am Universitätsklinikum Erlangen, Friedrich-Alexander Universität Erlangen-Nürnberg, Erlangen, Germany.

[6]School of Pharmacy, The University of Queensland, 20 Cornwall St, Woolloongabba, Queensland 4102, Australia.

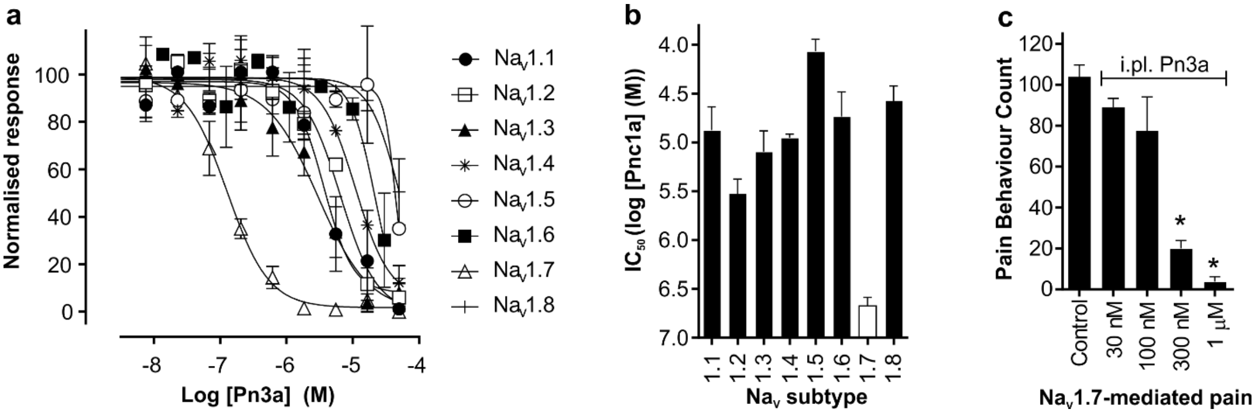

Supplementary Figure 1. *In vitro and in vivo activity of the Na$_V$1.7-selective inhibitor Pn3a. Na$_V$ subtype-selectivity of Pn3a was assessed using a high-throughput FLIPR^{Tetra} membrane potential assay in HEK293 cells heterologously expressing hNa$_V$1.1 – hNa$_V$1.8.* ***(a)*** *Representative concentration-response curve illustrating full inhibition of Na$_V$1.7-mediated responses at concentrations >300 nM) that do not affect any other Na$_V$ subtype substantially. n = 3 wells/concentration* ***(b)*** *IC_{50} (expressed as –log [concentration] (M), or pIC_{50}) values of Pn3a at hNa$_V$1.1 – hNa$_V$1.8. n = 3-5 independent experiments* ***(c)*** *To assess in vivo on-target effects of Pn3a, effects on pain behaviours after intraplantar injection of the selective Na$_V$1.7 activator OD1 (300 nM) were assessed as previously described (Deuis, Wingerd et al., 2016). Intraplantar administration of Pn3a (30 nM – 1 μM, 40 μl) significantly ($P<0.05$) reverses Na$_V$1.7-mediated pain behaviours. n = 3-12 animals/group. Data is presented as mean ± SEM. Statistical significance (*, defined as $P<0.05$) was determined using One-way ANOVA.*
